# A High-Resolution Map of Synteny Disruptions in Gibbon and Human Genomes

**DOI:** 10.1371/journal.pgen.0020223

**Published:** 2006-12-29

**Authors:** Lucia Carbone, Gery M Vessere, Boudewijn F.H. ten Hallers, Baoli Zhu, Kazutoyo Osoegawa, Alan Mootnick, Andrea Kofler, Johannes Wienberg, Jane Rogers, Sean Humphray, Carol Scott, R. Alan Harris, Aleksandar Milosavljevic, Pieter J de Jong

**Affiliations:** 1 BACPAC Resources, Children's Hospital of Oakland Research Institute, Oakland, California, United States of America; 2 Gibbon Conservation Center, Santa Clarita, California, United States of America; 3 Chrombios GmbH, Raubling, Germany; 4 Anthropology and Human Genetics, Department of Biology II, Munich University, Munich, Germany; 5 Wellcome Trust Sanger Institute, Wellcome Trust Genome Campus, Cambridge, United Kingdom; 6 Department of Molecular and Human Genetics, Baylor College of Medicine, Houston, Texas, United States of America; Fred Hutchinson Cancer Research Center, United States of America

## Abstract

Gibbons are part of the same superfamily (Hominoidea) as humans and great apes, but their karyotype has diverged faster from the common hominoid ancestor. At least 24 major chromosome rearrangements are required to convert the presumed ancestral karyotype of gibbons into that of the hominoid ancestor. Up to 28 additional rearrangements distinguish the various living species from the common gibbon ancestor. Using the northern white-cheeked gibbon (2n = 52) *(Nomascus leucogenys leucogenys)* as a model, we created a high-resolution map of the homologous regions between the gibbon and human. The positions of 100 synteny breakpoints relative to the assembled human genome were determined at a resolution of about 200 kb. Interestingly, 46% of the gibbon–human synteny breakpoints occur in regions that correspond to segmental duplications in the human lineage, indicating a common source of plasticity leading to a different outcome in the two species. Additionally, the full sequences of 11 gibbon BACs spanning evolutionary breakpoints reveal either segmental duplications or interspersed repeats at the exact breakpoint locations. No specific sequence element appears to be common among independent rearrangements. We speculate that the extraordinarily high level of rearrangements seen in gibbons may be due to factors that increase the incidence of chromosome breakage or fixation of the derivative chromosomes in a homozygous state.

## Synopsis

It is commonly accepted that mammalian chromosomes have undergone a limited number of rearrangements during the course of more than 100 million years of evolution. Surprisingly, some species have experienced a large increase in the incidence of rearrangements, including translocations (exchange between two non-homologous chromosomes), inversions (change of orientation of one chromosomal segment), fissions, and fusions. Within the primate order, gibbons exhibit the most strikingly unstable chromosome pattern. Gibbon chromosomal structure greatly differs from that of their most recent common ancestor with humans from which they diverged over 15 million years ago. The authors are interested in the mechanisms causing this extraordinary instability. In this study, they employed modern techniques to compare the human and white-cheeked gibbon chromosomes and to localize all the regions of disrupted homology between the two species. Their findings indicate that the molecular mechanism of gibbon chromosomal reshuffling is based on the same principles as in other mammalian species. To explain the 10-fold higher incidence of gibbon chromosomal rearrangements, it will be necessary to pursue future studies into other biological factors such as inbreeding and population dynamics.

## Introduction

During recent years, great progress has been made in understanding the evolutionary processes governing mammalian chromosomal organization. It is now commonly accepted that the mammalian karyotype has undergone a limited number of major rearrangements over the course of more than 100 million years [1]. A few species represent an exception to the rule by demonstrating a very high incidence of karyotypic changes. Mouse, rat, and dog are often cited as examples of exceptionally rearranged chromosomes compared to the putative ancestral mammalian karyotype [2–5] The small apes or gibbons (Hylobatidae) exhibit heavily reshuffled chromosomes relative to most other members of the primate order and, most significantly, relative to other members of the superfamily Hominoidea: the great apes and humans. Humans and great apes have a karyotype more similar to the ancestral mammalian karyotype, suggesting that the chromosomal instability evolved in the ancestor of the small apes. The high rate of karyotype rearrangement persisted from the common gibbon ancestor to the current species as indicated by the four karyomorphs that define the four gibbon genera: *Symphalangus* (siamang) 2n = 50, *Nomascus* (crested gibbon) 2n = 52, *Hylobates* (Hylobates group) 2n = 44, and *Hoolock* (hoolock gibbon) 2n = 38 [6–8]. The evolutionary mechanisms that generated this karyotype diversity may have terminated or may still be in action today.

Recent studies describing the dynamics of mammalian genome evolution indicate a “reuse” of genomic regions for independent evolutionary breakpoints in different lineages as well as the presence of hotspots and fragile sites more prone to rearrangements. These fragile loci frequently coincide with regions enriched for segmental duplications (SDs) in primates and involved in human genomic disorders [9–17]. Moreover, it is well known that transposable elements are responsible for chromosomal instability in *Drosophila* [18,19] and endogenous retroviruses are involved in genome shuffling in mammals [15,20,21].

Gibbon karyotypic changes have previously been investigated by cytogenetic banding analysis [6,22–24] and more recently by comparative chromosome painting [7,8,25–29] and reciprocal chromosome painting techniques [30,31]. The maps resulting from these experiments are limited by the resolution of fluorescence microscopy (about 3–5 Mb). As a result, it is difficult to correlate gibbon rearrangements detected by these methods with smaller scale genomic sequence features. A more detailed analysis of breakpoint regions may determine if the rearrangements are caused by gibbon-specific genomic sequence features. Alternatively, the breakpoints may coincide with sequence features found at rearrangement sites in other mammals, including human. To decide between these two alternatives, it is necessary to first map the numerous gibbon chromosome breakpoints at high resolution based on DNA sequence alignments and then to sequence the new junctions.

Here, we compared the karyotypes of *Nomascus leucogenys leucogenys* (NLE) (northern white-cheeked gibbon) and human using a combination of high-resolution genomic technologies: array comparative genome hybridization painting [32,33], bacterial artificial chromosome (BAC) end-sequence profiling [33], and confirmation by fluorescence in situ hybridization (FISH). Our approach made optimal use of BAC libraries from the human and gibbon genomes to create a map of 100 gibbon breakpoints relative to the human genome at a resolution of approximately 200 kb, the size of a typical BAC clone. We isolated 67 gibbon BAC clones spanning breakpoints with the intent of looking at the species-specific sequences in these regions. The full sequences of a subset of these clones provide insight into the architecture of rearranged chromosomal regions at the molecular level.

## Results/Discussion

### Overview of the Genomic Tools Used in the Experiments

The three main resources used in this study were 1) high-resolution microarray slides containing about 32,000 BAC clones spanning the entire human genome (“32K set”), 2) a genomic BAC library for the northern white-cheeked gibbon (CHORI-271) described in more detail at http://bacpac.chori.org/library.php?id=228, and 3) the latest genome assemblies of rhesus macaque (UCSC build rheMac2) and chimpanzee (UCSC build panTro1) used as outgroups to resolve rearrangements to the great ape or small ape lineage.

To better understand karyotype instability, gibbon-specific sequences at the break of synteny regions (BOSRs) need to be analyzed. To determine if it is possible to attribute breakpoints to specific sequence elements or to the genomic architecture of these regions, we sequenced a preliminary set of 11 gibbon BAC clones spanning BOSRs.

### Breakpoint Identification by Array Painting of Flow-Sorted Gibbon Chromosomes on Human Arrays

We employed array painting (see [32] and Materials and Methods for more details) to map the end-points of gibbon–human synteny regions relative to the human genome. In this technique the chromosomes carrying balanced reciprocal translocations are isolated by flow sorting, and the DNAs are labeled with two contrasting dyes (Cy3 and Cy5) and hybridized to an array of 32,000 human BAC DNAs spotted on a glass slide. A single hybridization thus permits the accurate mapping of breakpoints at a resolution determined by the genomic intervals between BACs on the array. A breakpoint detected by this method is referred to as a BOSR.

Optimal experimental conditions were found by hybridizing three gibbon chromosomes separately (NLE13, NLE20, and NLE10) in a preliminary array-painting experiment. Array-painting experiments were then economized by pooling the sorted gibbon chromosomes so that the gibbon chromosomes in each pool detected distinct human chromosomes. This “smart pooling” was possible because the gibbon–human synteny regions have previously been identified through chromosome painting [7,28,29,31]. Pooling allowed us to conduct array painting on four pools of gibbon chromosomes, which was equivalent to conducting 26 separate hybridizations for each gibbon chromosome (2n = 52). The pooling strategy is shown in Table S1.

**Figure 1:**
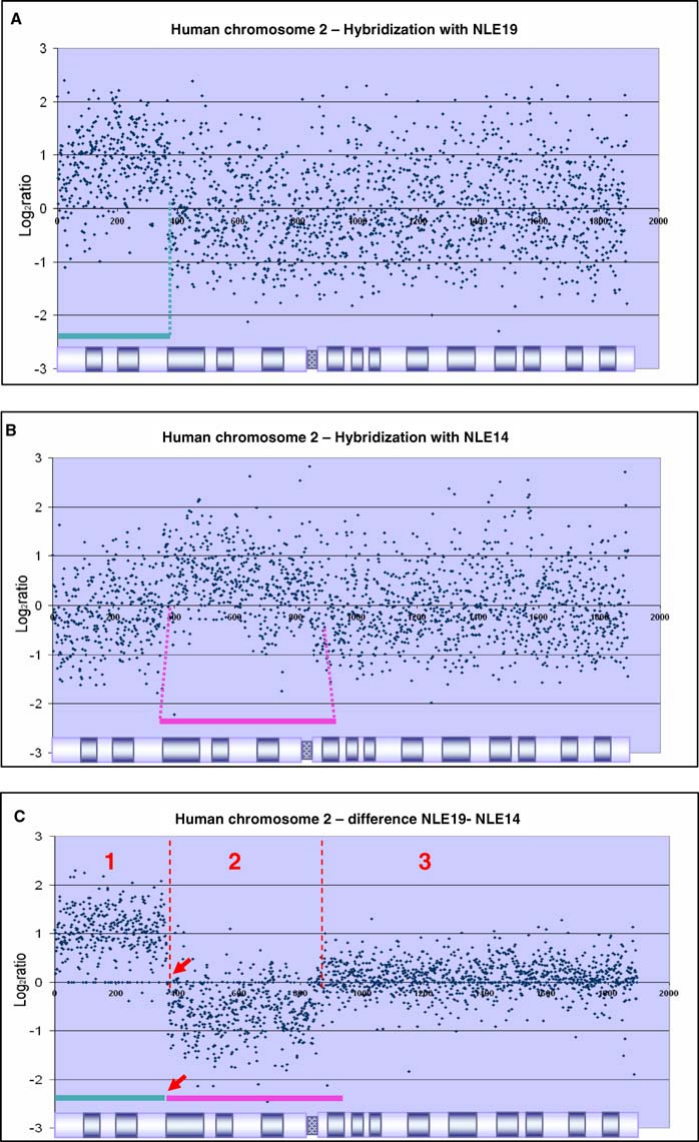
Identification of Break of Synteny Regions Using the Log_2_ Ratio Difference Method (A and B) The plotted value of Log_2_ ratio/chromosome length for human Chromosome 2 after hybridization with sorted gibbon chromosomes NLE14 and NLE19, respectively. (C) Results of the application of the difference method to the datasets in (A) and (B). After canceling out the systematic variation, it is possible to discern three different regions from left to right, one amplified (1), one deleted (2), and one at the baseline (3).

Single test and reference hybridization results revealed that the signal-to-noise ratio was too low for accurate detection of BOSR coordinates. After determining that the hybridization noise was systematic, we developed a noise-reduction method (Protocol S1) to obtain better definition of the breakpoints. Figure 1A and 1B shows the individual data plots for human Chromosome 2 obtained with pools containing flow-sorted chromosomes NLE14 and NLE19, respectively. These two gibbon chromosomes resulted from a reciprocal translocation and many inversions involving ancestral sequences homologous to human Chromosomes 2 and 17 [7]. After applying our noise-reduction method, we obtained a well-defined shift in the plateau values at a single location coinciding with the approximate site of the BOSR (Figure 1C). Results for all reciprocal BOSRs pairs are presented in Figure S1.

**Figure 2:**
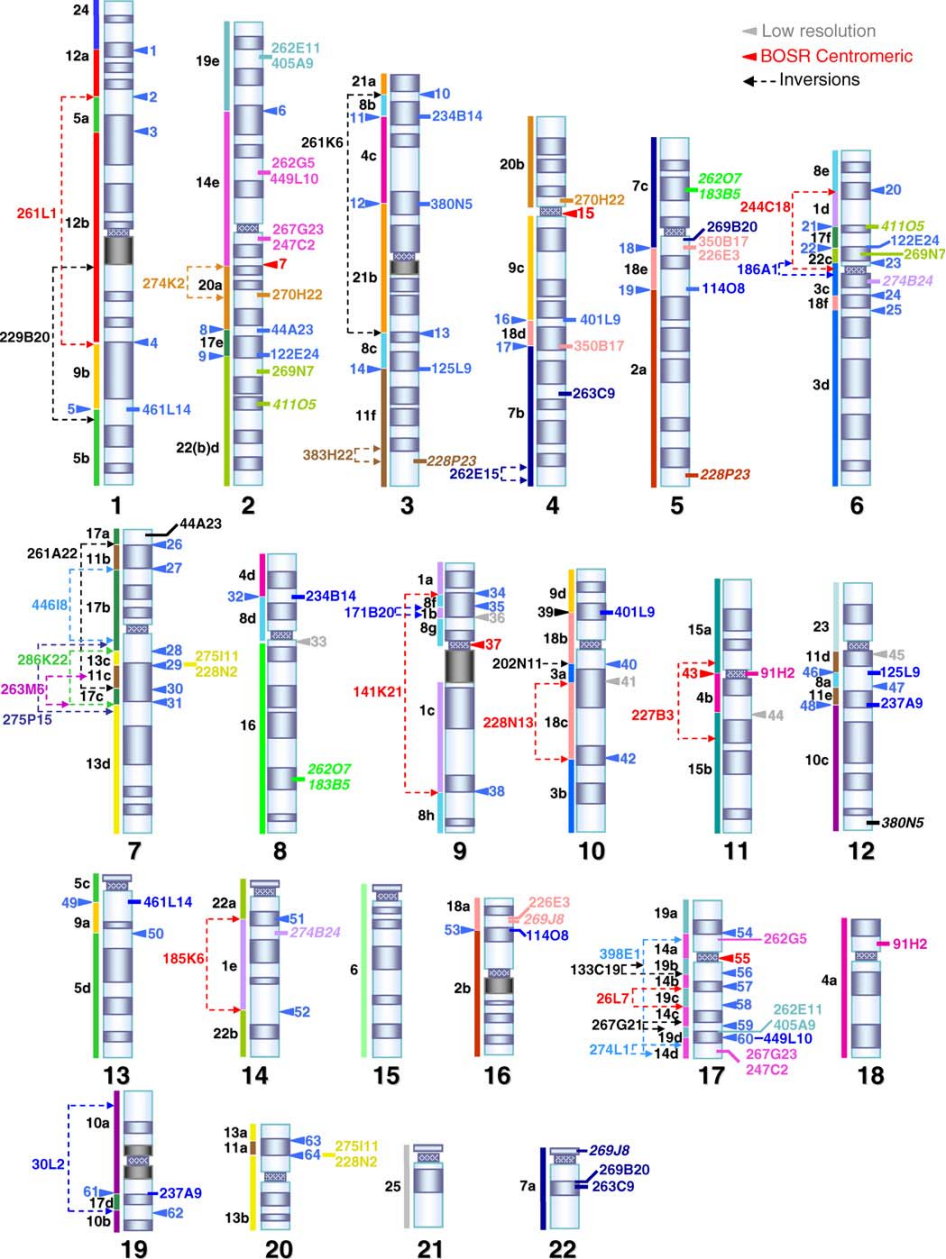
Comparative Map of Human and Gibbon Chromosomes Ideogram of human chromosomes with orthologous gibbon chromosomes identified by array painting represented by colored bars to the left of each chromosome. Each segment is named after the gibbon chromosome followed by a small letter that refers to its mapping order in the gibbon chromosome. The BOSRs have been defined for convenience by numbers (Table 1). Gibbon clones spanning breakpoints identified by BES mapping are also located on the map. Clones with map positions that disagree with the array-painting map are italicized.

Altogether, 64 BOSRs have been mapped to the human genome (Hg17, UCSC May 2004) (Table 1 and Figure 2). Among these 64 regions, four correspond to human centromeres and one overlaps with the site where two ancestral ape chromosomes fused telomere-to-telomere to form human Chromosome 2 (2q13-2q14). Six noncentromeric BOSRs (BOSR-33, -41, -44, -45, -52, and -61) could not be mapped with a precision higher than 850 kb. In the case of BOSR-33 and BOSR-45, mapping resolution was affected by their pericentromeric locations where abundant human genomic duplications caused noisy plots. In the particular case of BOSR-44, we confirmed that the high noise resulted from a neighboring keratin-associated protein gene cluster located on human chromosome 11q13.5 (data not shown).

**Table 1:**
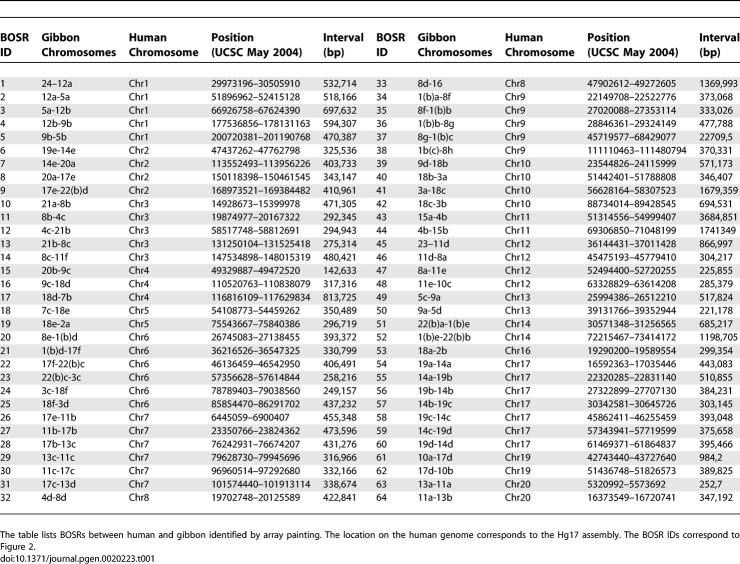
Array-Painting Results

### FISH Confirmation and Cross-Species Analysis of BOSRs

**Figure 3:**
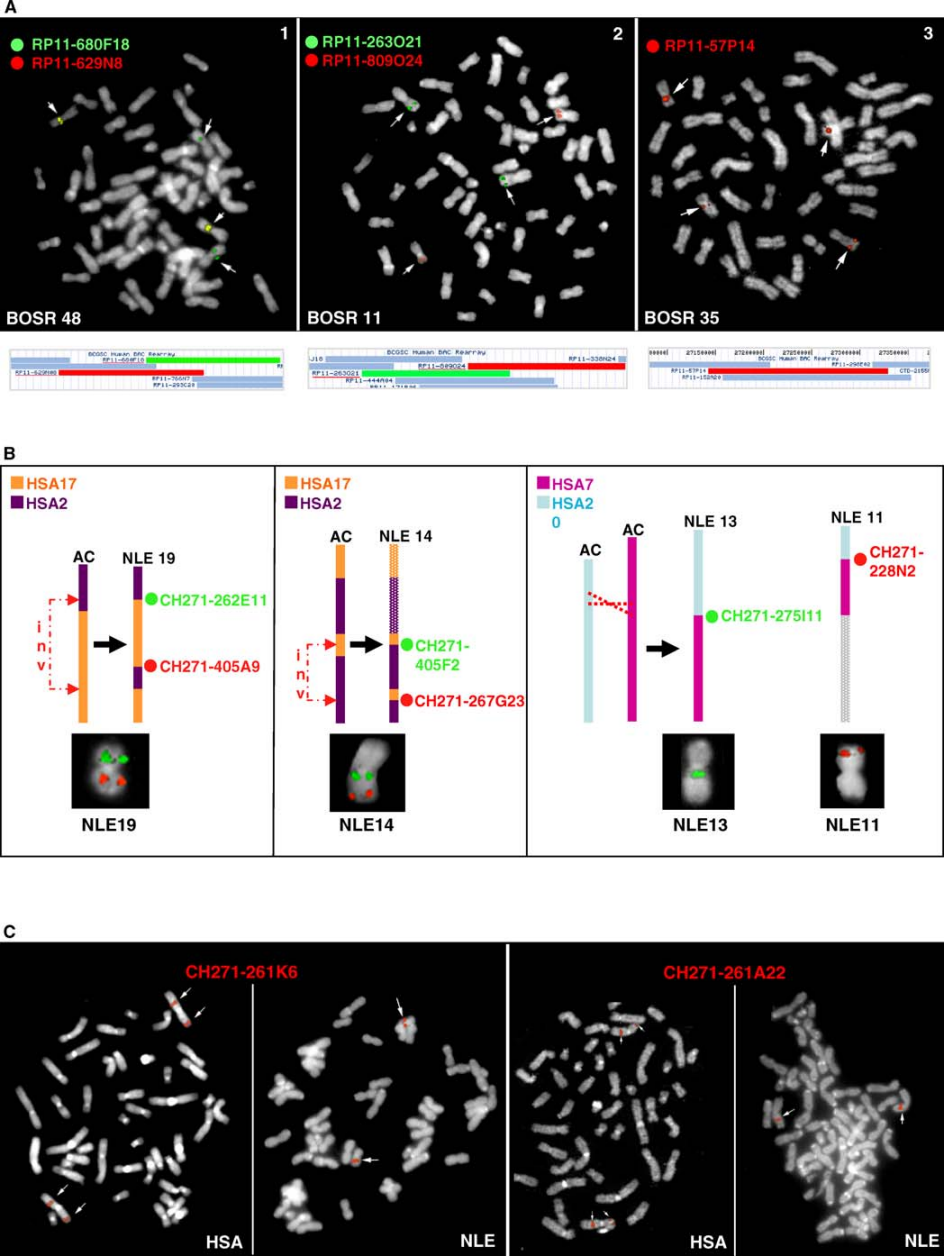
Breakpoint Validation by FISH (A) FISH experiments to validate breakpoints identified by array painting. Images 1 and 2 show hybridization on NLE metaphase preparations with human BACs spanning breakpoints identified by array painting. The yellow color in image 1 is due to the overlap of red and green spots as both BACs map on the same chromosome. Image 3 shows a similar experiment done on HLA metaphase preparations. The reciprocal position of the BACs used in each experiment is shown in the boxes below the images. (B) FISH validation experiments on six gibbon BAC clones spanning three reciprocal breakpoints for the same rearrangement. In the diagrams, the rearrangements are illustrated starting from the ancestral chromosome form. Abbreviation: AC, ancestral chromosome. (C) Gibbon BACs spanning inversion breakpoints were tested by FISH on human and gibbon metaphases. A BAC spanning an inversion in gibbon is expected to give a split signal on the human chromosome and a single signal on the corresponding gibbon chromosome.

In order to confirm the accuracy of array-painting results, ten BOSRs were validated by FISH experiments where BAC clones from the “32K set” were hybridized to NLE metaphase preparations. In most of the cases, a single BAC hybridized to two disparate locations as expected. Only BOSR-32 and BOSR-11 demonstrated a single hybridization signal, suggesting that the breakpoint was located between two BACs or in a small region of overlap between them. A few examples of FISH experiments are shown in Figure 3A.

Additionally, six of these BOSRs, thought to be common to all gibbons [7,29], were mapped to the gibbon species Hylobates lar (HLA) (2n = 44) using the same human FISH probes (Figure 3A). In five cases (BOSRs −8, −16, −19, −35 and −53), the human BACs produced similar split signals on HLA and NLE metaphase preparations, indicating that the breakpoint is shared by the two species, strengthening the original hypothesis that the breaks occurred in the karyotype of a common ancestor [7,29]. However, in one case (BOSR-6), we did not observe a split FISH signal on the HLA metaphase preparations (data not shown). This result suggests that this translocation occurred in the NLE lineage after the split from the common ancestor of NLE and HLA.

### Breakpoint Identification by Gibbon BAC End Sequence Mappings onto Human

Both ends of 5,376 clones from the gibbon genomic BAC library CHORI-271 were sequenced and mapped onto the human genome using the BLAT program [34] (Protocol S1). Gibbon BACs spanning putative breakpoints were identified using the paired BAC end-sequence mappings to the human genome by applying a “pairing criteria” implemented as a script (Protocol S1). Mapping results are summarized in Table 2.

**Table 2:**
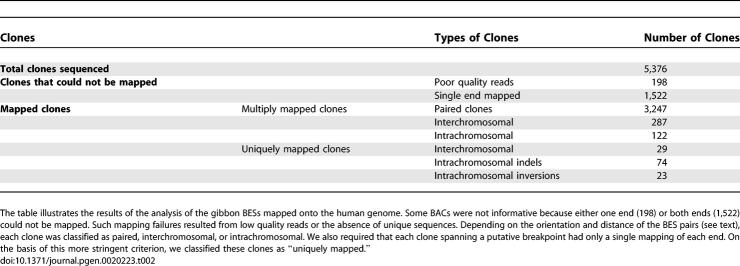
Analysis Using the Pairing Criteria of Gibbon BES Mapped on the Human Genome

For the purpose of visualizing the gibbon clones mapped onto the human genome, we created a software tool that graphically depicts the chromosomal position of gibbon BAC mappings on the human genome. This software allows for full chromosome views as well as views showing a user-configurable window size (Protocol S1 and Figure S2A and S2B). This tool allowed us to easily overlay the BAC end sequence (BES) mappings onto mappings obtained from array painting and manually identify additional clones.

### Interchromosomal Rearrangements

Gibbon clones identified as putatively spanning interchromosomal rearrangements were mapped by FISH on gibbon metaphase preparations. Clones giving signals on more than two gibbon chromosomes were considered possible clone artifacts (chimeric clones) or duplicated regions in the gibbon and were removed from further analysis. We also screened the gibbon BAC CHORI-271 library (Materials and Methods) in order to identify at least two additional clones spanning identified breakpoints. Table 3 reports clones that were validated by combining the array-painting, FISH, and library-screening approaches. Twenty-five clones were confirmed by additional overlapping clones with BES mappings to the same pair of human chromosomes. In seven instances, we identified clones spanning reciprocal breakpoints by library screening and subsequent BES. These clones map on the same region of the human genome; however, they are localized on two derivative gibbon chromosomes in the case of translocations, or different regions of the same chromosome in the case of inversions. We verified that these clones carry reciprocal breakpoints by FISH hybridization on gibbon metaphases. Examples of FISH experiments are shown in Figure 3B.

**Table 3:**
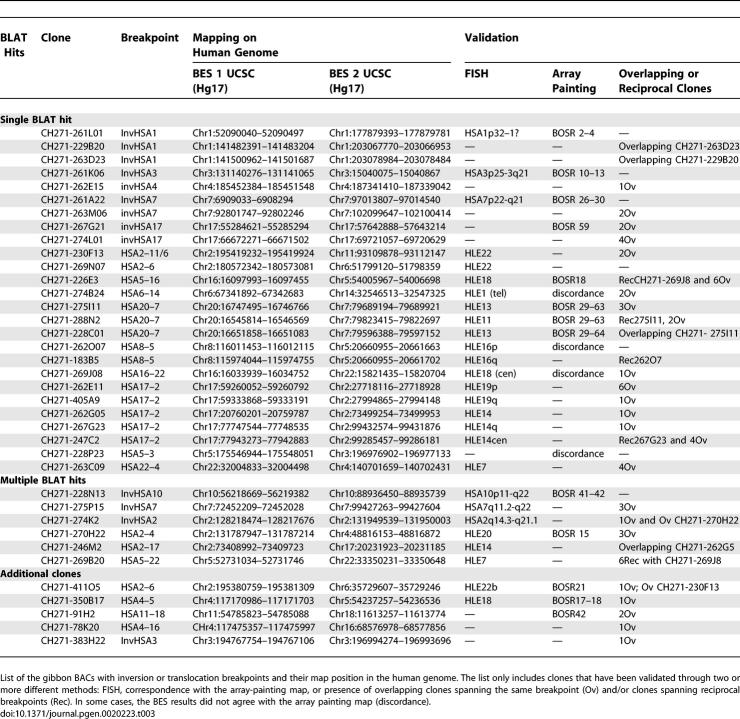
Gibbon BAC Clones Spanning Breakpoints Identified by Mapping End Sequences

### Intrachromosomal Rearrangements

Based on our pairing criteria, most of the clones identified as spanning intrachromosomal rearrangements resulted from insertion/deletions (indels) in either the gibbon or the human genomes (74 out of 97). Indels cause discrepancies between paired BES mapping distances on the human genome compared to the average gibbon BAC insert size. We formally defined this as BES mappings at a distance less than or greater than three standard deviations from the 172-kb-clone insert size. We verified the insert size of the 74 clones spanning putative indels using NotI digestion and pulsed-field electrophoresis. Based on the pulsed-field electrophoresis, the 60 gibbon BACs with BES mapping distances of 40–60 kb relative to the human genome were found to not be indels, as they had actual insert sizes in the 40–60 kb range. We presumed that the remaining 14 clones with BES distances exceeding 300 kb represent actual insertions in the human genome or deletions in the gibbon genome. Results are summarized in Table 4.

**Table 4:**
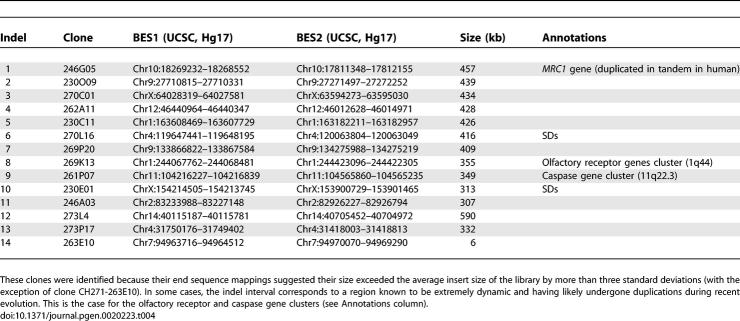
Gibbon BAC Clones Spanning Indels

Clones putatively spanning inversion breakpoints were validated by comparisons with BOSRs previously defined by array painting (Table 3). Large-scale inversions were further confirmed by hybridizing the gibbon clones onto human metaphase preparations (Figure 3C). Additional clones spanning the same breakpoint were obtained by screening the CHORI-271 library as described for interchromosomal breakpoints. Through this validation process, 15 out of 23 clones were confirmed and the remaining eight clones were removed from consideration.

### Combination of Array Painting and End-Sequencing Mapping

The goals of our study were 1) to obtain a map of the BOSRs between human and gibbon at high resolution and 2) to identify species-specific clones spanning chromosomal rearrangements for use in further molecular analysis. In pursuit of our second goal, we selected for further analysis 38 gibbon BAC clones corresponding to BOSRs identified by array painting human BACs. We constructed probes across these BOSRs at 75-kb intervals based on the human genome sequence and used these probes to screen the gibbon library. Using this approach, we identified an additional 26 gibbon clones containing breakpoint loci (15 inversions and 11 translocations) (Table 5).

**Table 5:**
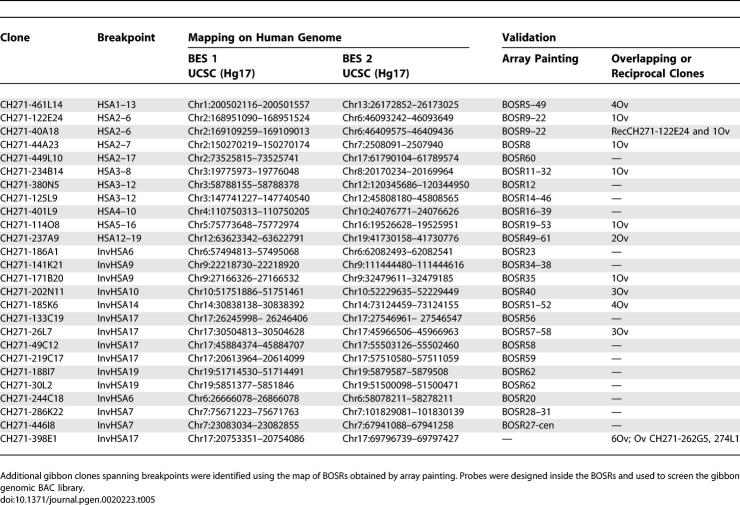
Gibbon BAC Clones Isolated Using the Array-Painting Map Combined with Filter Screening and BES Mapping

### Identification of Breakpoints Specific to the Gibbon Lineage

To ensure that we identified rearrangements that occurred in the gibbon lineage, we mapped the BES of gibbon clones identified as spanning rearrangement breakpoints onto the latest genome assemblies of rhesus macaque (UCSC Build rheMac2) and chimpanzee (UCSC Build panTro1) using BLAT. We removed ambiguous mappings and classified the remaining mappings using the same pairing criteria applied to the human genome mappings. We classified putative rearrangements as 1) gibbon specific if gibbon was rearranged relative to human, chimpanzee, and macaque; 2) great ape specific if gibbon was not rearranged relative to macaque, but was rearranged relative to human and chimpanzee; and 3) human specific if gibbon was not rearranged relative to macaque and chimpanzee but was rearranged relative to human (Table S2). Based on this classification, we identified three human-specific rearrangements and four great ape–specific rearrangements.

One of the events classified as great ape–specific is the inversion of human Chromosome 3, with breakpoints at 3p25 and 3q21, which are regions already known as rearrangement hotspots in primates [35]. BOSR-10 and BOSR-13 from the array-painting map span these inversion breakpoints, and clone CH271-261K6 spans one of the breakpoints. Additionally, we identified a clone spanning the breakpoint of an inversion that occurred in the ancestor of the great apes in the chromosome homologous to human Chromosome 7. Müller et al. [36] previously described this inversion, which occurred in the lineage leading to human and African great apes. Human, chimpanzee, and gorilla therefore share the same derivative form, while orangutan, small apes, and macaque share the ancestral one.

In total, we identified 110 breakpoints between human and gibbon chromosomes due to intra- and interchromosomal rearrangements. Of those, 100 occurred during the evolution of the gibbon.

### Analysis of Gibbon-Specific Breakpoints Spanning Regions on the Human Genome

It is widely accepted that regions of chromosomal instability and SDs colocalize more frequently than expected by random chance. SDs are blocks of DNA 1–400 kb in length, repeated in the genome with a high level of sequence identity (>90%) [37]. The association between SDs and evolutionary breakpoints in primates has been repeatedly reported [13,14,16,36,38], leading to speculation that these large blocks of homology predispose the flanking regions to rearrangement by nonallelic homologous recombination. Obviously, one would like to explore correlations between gibbon SDs and chromosomal rearrangements. Since the gibbon genome has not been sequenced, we used the human genome as the most closely related surrogate. Our assumption is that chromosomal regions enriched for SDs in gibbon may also show an enrichment of SDs in the human genome.

We first analyzed the overlap between the human regions orthologous to gibbon-specific BOSRs and the human SDs reported in the UCSC browser [39]. We found that 46 of 100 regions (46%) overlapped with at least one SD. This correlation remained strong (42%) when breakpoints located in the centromeric regions and regions identified with lower resolution were removed from the analysis. It is important to note that the BOSRs represent segments that are, on average, 220 kb. Therefore, the SD may overlap with the BOSR, but not necessarily include the actual breakpoint.

**Figure 4:**
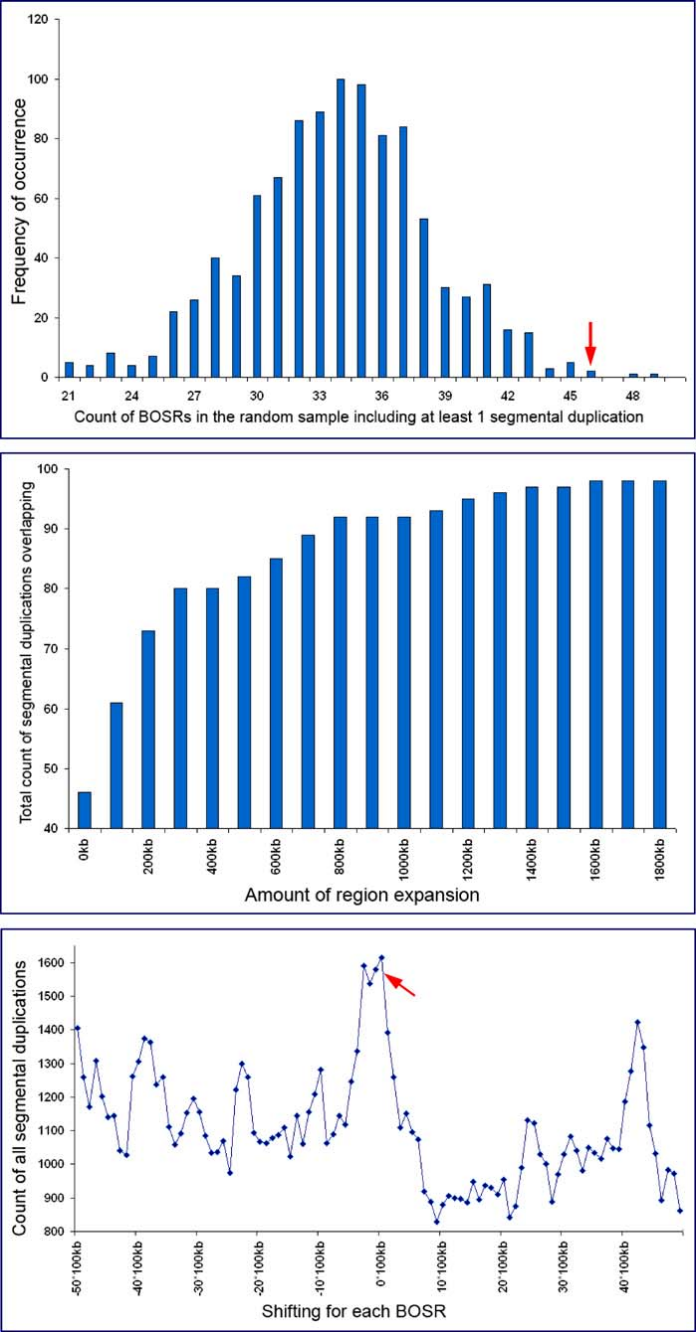
Association of the Breakpoint Regions with Segmental Duplication (A) The figure shows the sampling distribution of the overlap between SDs and a random set of regions obtained by relocating our original sample 1,000 times in the corresponding chromosomes. The original sample fell more than three standard deviations away from the mean of the simulated distribution (red arrow). (B) The regions from the original sample were expanded in 100 kb increments. The number of regions overlapping with SDs at each step is shown. (C) We measured the amount of overlap (in base pairs) of our 100 regions, while shifting each of them up to 5 Mb left and right of their original positions in 100 kb increments. The strong correlation between the original position (red arrow) and SD content is shown.

To statistically validate the significance of these data, a simulation was run in which the 100 breakpoint regions were randomly relocated 1,000 times in their original chromosome, emulating a random-breakage model (Protocol S1). The result was a count of the number of regions overlapping SDs at each step of the simulation. The association of 100 detected breakpoint regions with SDs fell more than three standard deviations away from the mean of the simulated sampling distribution (Figure 4A), indicating that this association is highly unlikely to have occurred by chance.

Measuring overlap alone does not sufficiently express the proximity of these regions to SDs; thus, the breakpoint regions were expanded in 100 kb increments and monitored for variation in the number of regions overlapping with SDs (Figure 4B). Out of the 100 regions, 80 overlapped with SDs after being expanded by 500 kb and 97 after being expanded by 1.5 Mb. We confirmed that such an overlap was unlikely to occur by chance by simulating randomly relocated regions expanded in a similar manner (data not shown). Finally, the association between our breakpoints and SDs was examined by measuring their base-pair overlap while shifting the breakpoint regions up to 5 Mb upstream and downstream of their original positions using 100 kb increments. The overlap was highest when the breakpoints were in their original positions and overlap progressively decreased with an increase in the distance shifted (Figure 4C).

Of the 46 BOSRs overlapping with human SDs, 27 are covered by at least one gibbon clone. We used these clones for interphase FISH experiments on NLE. In 22 cases, multiple signals were evident on NLE nuclei, suggesting duplicated regions. The remaining five clones showed no indication of duplications at the cytogenetic level. Additionally, 10 out of these 22 clones were duplicated in two other species of gibbon (Symphalangus syndactylus and HLA) belonging to different genera. These data suggest that the SDs most likely appeared within a common ancestor (Figure S3).

We cannot assume that these duplications were responsible for the chromosomal rearrangement events in NLE, as we have insufficient data to indicate the duplications predated the breakage events. However, this correlation is consistent with a well-established model in which duplications are indicative of the “plasticity” of a region [21].

### Analysis of Fully Sequenced Gibbon BACs Spanning Rearrangement Breakpoints

We analyzed the finished sequence of 11 gibbon clones comprising a representative sample of the clones identified in this study. Although the rearrangement events occurred in ancestral chromosomal sequences that are in part altered in the current genome, the study of orthologous sequences can nevertheless still provide us with information about the nature of the genomic instability present in these regions. The breakpoint spanned by each clone was localized to the break of synteny between the gibbon clone and the human genome.

**Figure 5:**
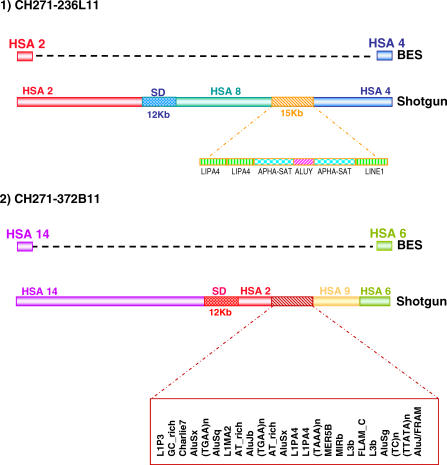
Analysis of Fully Sequenced Gibbon BAC Clones Two sequenced gibbon clones spanning rearrangement breakpoints revealed the presence of additional segments of synteny not observed by other methods. In both cases, the first break of synteny was found to contain SDs and the second to contain interspersed repeats.

The first interesting discovery to emerge from the analysis of sequenced clones was the presence of “micro-rearrangements” that fell below the resolution of the BES mappings. Micro-rearrangements were observed in two clones, CH271-372B11 and CH271-236L11, in which the complete sequence revealed regions orthologous to human chromosomes other than those predicted by BES or array painting (Figure 5). In both cases, one of the breakpoints was found to be enriched for SDs, while the other breakpoint fell within an interspersed repeat-rich region (Figure 5). This finding suggests that the gibbon genome might be more rearranged than previously observed. Our sample therefore contained 15 breaks of synteny to be analyzed rather than 11.

Five BOSRs, including the two BOSRs mentioned above, were within 5 kb of SDs. One example is the clone CH271-262E11 mapping to NLE Chromosome 19 and spanning a breakpoint between human Chromosomes 2 and 17. The BOSR in this clone was identified at the base-pair level and was found to be adjacent to the growth-hormone gene cluster. The breakpoint is 20 bp away from a duplicated segment containing the ortholog of gene *GH2*. At an approximate 2-kb distance from *GH2*, we found a block of about 15 kb duplicated in tandem. This block corresponds to a SD in the human genome located at Chr17:59292722–59308474 that is repeated in the nearby genomic region Chr17:59316565–59331044 (Hg17, UCSC May 2004). These duplications contain a second gene from the growth hormone cluster (*CHS2*). It has been shown [40] that the growth hormone family experienced an enhanced rate of duplication in primates compared to other mammals, with many duplication events occurring before the divergence of Old World monkeys and New World monkeys. Furthermore, Ye et al. [40] recently showed that the NLE growth hormone cluster behaved differently from other primates, with rapid evolution occurring after the divergence of the gibbon ancestor from the great apes. The coexistence of duplications and an inversion breakpoint indicate this region is highly unstable and may be one of the rearrangement hotspots of primate genomes.

Six of the BOSRs coincided with interspersed repeats (SINEs, LINEs, and LTRs). In clone CH271-236L11, an alpha-satellite was identified due to the proximity of the BOSR to a centromere (Figure 5). Clones CH271-228C1 and CH271-86M19 span the reciprocal breakpoints of a translocation between human Chromosomes 7 and 20. In both clones, an (AT)n repeat separates the two homologous segments, preventing us from localizing the junction to the base-pair level. The (AT)n repeat in clone CH271-228C1 is 300 bp, and in CH271-86M19 it is 168 bp. A simple repeat classified as “AT rich” is also present in the corresponding position of human Chromosome 7. We confirmed the ancestral origin of this repeat by PCR amplifying and sequencing the orthologous region in gibbons belonging to three additional genera (*Hylobates agilis, Bunopithecus hoolock,* and *S. syndactylus*). We could therefore exclude the possibility that the repeat was a consequence of the rearrangement.

The presence of the AT-rich repeat in relation to these BOSRs may indicate a different breakpoint-inducing mechanism. Recently, Gotter and colleagues showed that the propensity to form secondary structures such as stem-loops can confer fragility to DNA [41]. Using the M-Fold sequence analysis package (http://bioweb.pasteur.fr/seqanal/interfaces/mfold-simple.html), we confirmed that the AT-rich repeats from the three clones give rise to long stem-loop structures (data not shown). At this point we do not have enough data to assume that this was the mechanism responsible for the translocation occurring during the evolution of the *Nomascus* genus. However, the cooccurrence of an evolutionary breakpoint and an AT-rich repeat region is intriguing.

### Conclusion

This study describes the mapping and validation of a large number of syntenic breakpoints between homologous chromosomes of human and NLE. All the translocation breakpoints previously identified by chromosome-painting studies were mapped to the human genome at a greatly increased resolution. About 20 additional rearrangements were discovered as a result of the higher sensitivity of our approaches. Overall, our research identified about 100 breakpoints occurring in the gibbon lineage. The study also yielded gibbon BACs containing breakpoint sites. In 11 sequenced gibbon BACs, we found elements near the breakpoints previously shown to play a key role in primate chromosome plasticity and evolution. Within the sequenced BACs three different patterns were evident. First, two BACs contained additional breakpoints that may have resulted from a complex, nonreciprocal translocation event or from subsequent chromosomal rearrangements. Recent high-resolution breakpoint analyses on human translocations thought to be balanced showed various “microtranslocations” [42]. Thus, translocations in human pathology and primate evolution may not always be simple breaks involving just two chromosomes but may be more complicated. Second, a correlation between SDs and evolutionary breakpoints in primates and other mammals has previously been suggested [11,13,16,43]. When all 100 gibbon-specific breakpoints were analyzed, a strong enrichment for SDs (in the human genome) was observed within 200 kb of the actual breakpoints (in the gibbon genome). Third, interspersed repeats have been linked to genomic instability in other studies, and several evolutionary breakpoints in primates are known to have occurred in repeat-rich areas [21,38,44,45]. Two of the sequenced BACs contained a breakpoint immediately adjacent to an AT-rich repeat with the potential to form stem-loop structures [41].

No generalized pattern unique to gibbon breakpoints is evident from the present molecular data. It remains to be determined if the greater number of chromosomal rearrangements in the small apes is due to an enhanced frequency of chromosomal breakages or an increased ability to rescue derivative chromosomes in comparison to other mammals, possibly due to mating behavior or inbreeding. We believe that these questions may be answered by examining additional aspects of small ape biology such as behavioral factors and population dynamics.

## Materials and Methods

### Array painting.

BACs (32,855) spanning 95% of the human euchromatic genome were assembled and rearrayed into 384-well microtiter dishes [46,47]. DNA was purified, amplified using the DOP (degenerate oligonucleotide-primed) PCR method, and spotted on CMT-GAPS UltraGaps coated glass slides (Corning, www.corning.com). Gibbon chromosomes were sorted on a FACS Vantage flow cytometry system (BD Biosciences, www.bdbiosciences.com) as described for previous experiments [31]. The NLE lymphoblast cell line used for chromosome sorting was the same as described by Müller et al. [31] and Schröck et al. [48]. Karyotype analysis revealed homozygosity for a known translocation polymorphism involving gibbon Chromosomes 1 and 22 (forms 1b and 22b). The only culture artifact observed was a trisomy for gibbon Chromosome 14. However, our karyotype analysis does not exclude the possibility that a small fraction of rearrangements observed by array painting were caused by cell-culture artifacts. DOP-amplified DNA [49] from flow-sorted chromosomes were subjected to a secondary DOP PCR and labeled with Cy3-dUTP (Amersham Biosciences, www5.amershambiosciences.com). Chromosomes X and Y were not included in this analysis because these are not involved in gibbon evolutionary translocations based on chromosome-painting studies. Anonymous human reference DNA was obtained from Children's Hospital Oakland Research Institute and amplified by DOP PCR. Labeling and hybridization were performed essentially as described by [50]. Hybridization images were generated by scanning the slides on a 4000B scanner (Axon Instruments, http://www.moleculardevices.com). The images were first processed using GenePix Pro 5.1 (Axon Instruments). The primary experimental data (GenePix Results files) were subjected to fully standardized data analysis (flagged spots removal, background subtraction, and loess normalization) by uploading them to the BioArray Software Environment microarray analysis software installation [51], which performs standard normalization. The final output was human chromosome specific plots of Log_2_ ratio values versus chromosome location as well as a whole-genome view.

### FISH.

FISH was used as a validation method for BOSRs identified uniquely by array painting or for gibbon BACs spanning inversions. Metaphase preparations of NLE were obtained from the same cell line used for chromosome sorting and previously described by Müller et al. [31] and Schröck et al. [48]. The cell lines used for metaphase preparations of HLA and S. syndactylus were the ones described by Jauch et al. [25] and Koehler et al. [26], respectively. *Homo sapiens* (HSA) metaphase preparations were prepared from peripheral blood culture.

BAC DNA extraction was performed as reported by Ventura et al. [25]. FISH experiments were performed essentially as described by Lichter et al. [52]. BACs were labeled either with biotin-dUTP or digoxigenin-dUTP by standard nick-translation assay. Fluorescent signals were obtained using avidin-FITC (Vector Laboratories, www.vectorlabs.com) and anti-digoxigenin-rhodamine antibodies (Roche, http://www.roche-diagnostics.com). When confirming translocation breakpoints on gibbon chromosomes, BACs were hybridized together with chromosome-specific painting probes obtained from sorting lymphoblastoid and somatic hybrid cell line chromosomes followed by DOP PCR [52]. Digital images were obtained using a Zeiss Axioskop (Carl Zeiss Inc., www.zeiss.com) microscope equipped with a CCD-1300DS (VDS Vosskuehler GmbH, www.vdsvossk.de) or a SenSys (Photometrics, www.photomet.com) cooled CCD camera. Pseudocoloring and merging of images was performed using SmartCapture (Digital Scientific, www.digitalscientific.co.uk) or Adobe Photoshop software (Adobe Systems Inc, www.adobe.com).

### Library screenings.

To identify BACs spanning putative breakpoint regions, overgo probes of 40 bp [53] were designed from end sequences of selected gibbon BAC clones. To search for reciprocal breakpoints, the overgo probes were designed from the human sequence at 170 kb from the BES location. All the probes were pooled together and hybridized to high-density filters of the CHORI-271 library following procedures already described [54]. Subsequently, the positive clones obtained from this first screening were rearrayed on small filters. Each small filter was used for hybridization with individual probes.

The images were analyzed with the software ArrayVision Ver6.0 (Imaging Research Inc., www.imagingresearch.com).

## Supporting Information

Figure S1 Identification of BOSRs between Human and Gibbon Chromosomes by Array Painting The results of array painting experiments done with different pools were combined for each human chromosome. After applying the difference method for noise reduction (see text) we identified all 64 BOSRs at a resolution of 300 kb (average) with the employment of a limited number of experiments. The figure shows the results obtained for all human chromosomes.
(240 MB PPT)

Figure S2 Translocations Viewer This tool was developed in order to easily localize gibbon clones spanning a translocation or inversion breakpoint in human. Figure S2A corresponds to a full genome view and Figure S2B corresponds to Chromosome 16. Gibbon clones are represented by the blue arrows, taking into account the orientation of each BES. On the bottom of the window is a density plot of human SDs.
(493 KB PPT)

Figure S3 Interphase FISH Experiments to Show Duplications in Gibbon A sample of gibbon BAC clones overlapping human SDs was hybridized on NLE nuclei. The presence of duplications was revealed by the presence of either multiple signals or a single but broadened signal. The figure shows the results obtained with four clones also tested on HLA and S. syndactylus.
(122 KB JPG)

Protocol S1 Click here for additional data file. (41 KB DOC)

Table S1 Composition of Gibbon Sorted Chromosome Pools for Array-Painting Experiments The gibbon chromosomes were divided into four pools in order to minimize the number of array-painting experiments. A smart-pooling strategy was used, taking advantage of the data available in the literature. Through this approach the repetition of one human chromosome in the same pool was avoided. Additionally, three gibbon chromosomes were hybridized in individual experiments.
(45 KB DOC)

Table S2 Comparative Mapping of Gibbon Clones Spanning Breakpoints on Rhesus Macaque and Chimpanzee Genome Assemblies The table reports the outcome of the mapping of gibbon clones spanning BOSRs on the latest genome assembly of Rhesus macaque (reMach2) and chimpanzee (panTro1). Depending on the result, clones were classified into three evolutionary groups: 1) gibbon specific, 2) great ape specific, and 3) human specific.
Mapping results not consistent with human are in italics.
(134 KB DOC)

### Accession numbers

The National Center for Biotechnology Information (NCBI) Entrez Gene (http://www.ncbi.nlm.nih.gov/entrez/query.fcgi?db=gene) accession numbers for the genes discussed in this paper are *CASP1* (NM_033294), *CASP4* (837), *CASP5* (838), *GH2* (2689), *KRTAP5–10* (387273), *KRTAP5–11* (440051), *KRTAP5–7* (440050), *KRTAP5–8* (57830), *KRTAP5–9* (3846), *MRC1* (4360), *OR11L1* (391189*),OR13G1* (441933), *OR1C1* (26188), *OR2G2* (81470), *OR2G3* (81469), *OR5AT1* (284532), and *OR6F1* (343169).

## Abbreviations

BAC: bacterial artificial chromosome
BES: BAC end sequence
BOSR: break of synteny region
DOP PCR: degenerate oligonucleotide-primed PCR
FISH: fluorescence in situ hybridization
HLA: *H. lar*
HSA: Homo sapiens
indel: insertion/deletion
NLE: *Nomascus leucogenys leucogenys*
SD: segmental duplication
