# Supplementary material for: A High-Resolution Map of Synteny Disruptions in Gibbon and Human Genomes
Source: PLoS Genet. 2006 Dec 29;2(12):e223. doi: 10.1371/journal.pgen.0020223 (PMC1756914; doi:10.1371/journal.pgen.0020223)
Supplement: Figure S1 — The results of array painting experiments done with different pools were combined for each human chromosome. After applying the difference method for noise reduction (see text) we identified all 64 BOSRs at a resolution of 300 kb (average) with the employment of a limited number of experiments. The figure shows the results obtained for all human chromosomes. (240 MB PPT) [file pgen.0020223.sg001.ppt]

## Slide 1
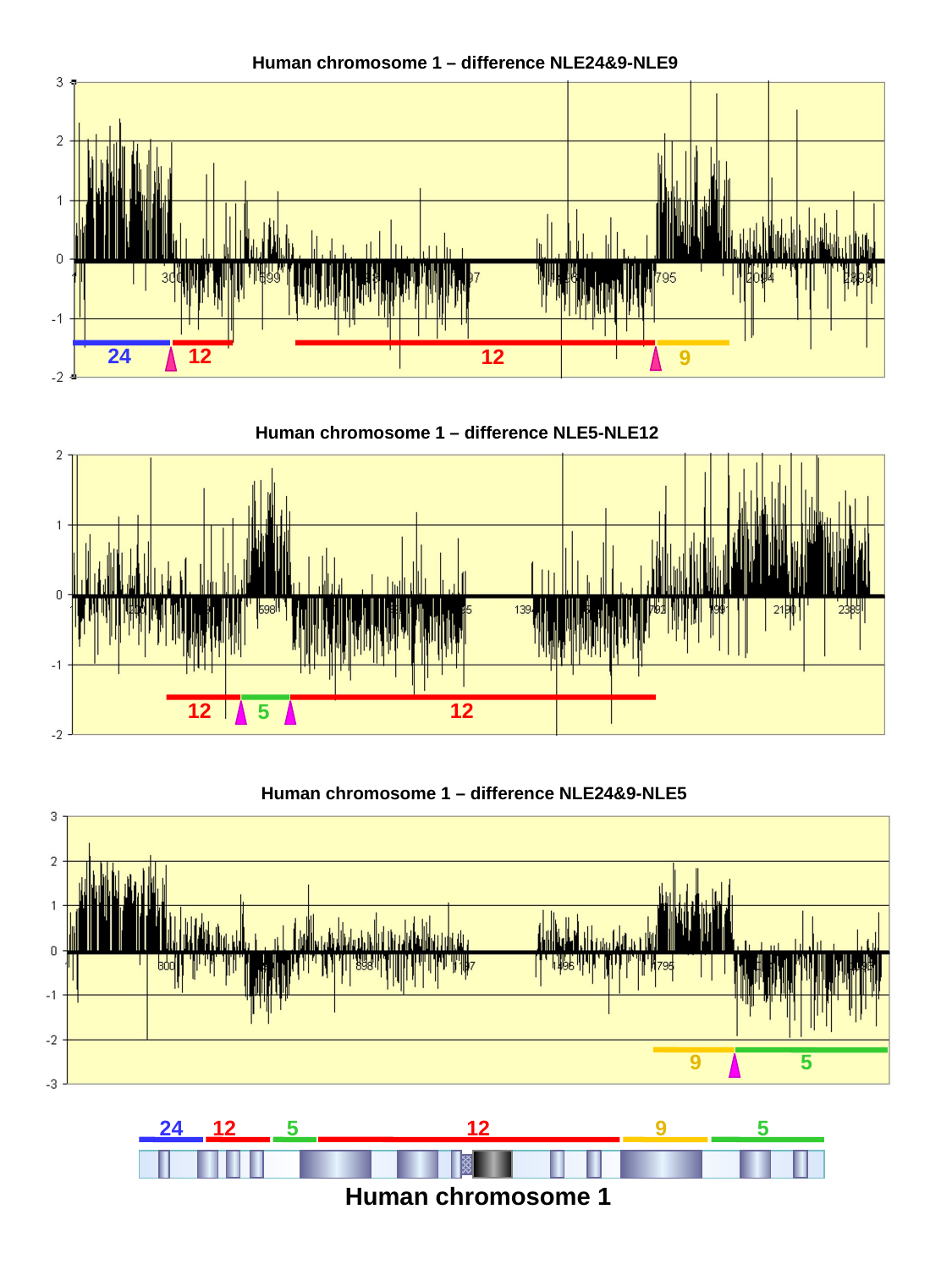

Human chromosome 1 – difference NLE24&9-NLE9
24
12
12
9
Human chromosome 1 – difference NLE5-NLE12
12
12
5
Human chromosome 1 – difference NLE24&9-NLE5
9
5
24
12
12
5
5
9
Human chromosome 1

## Slide 2
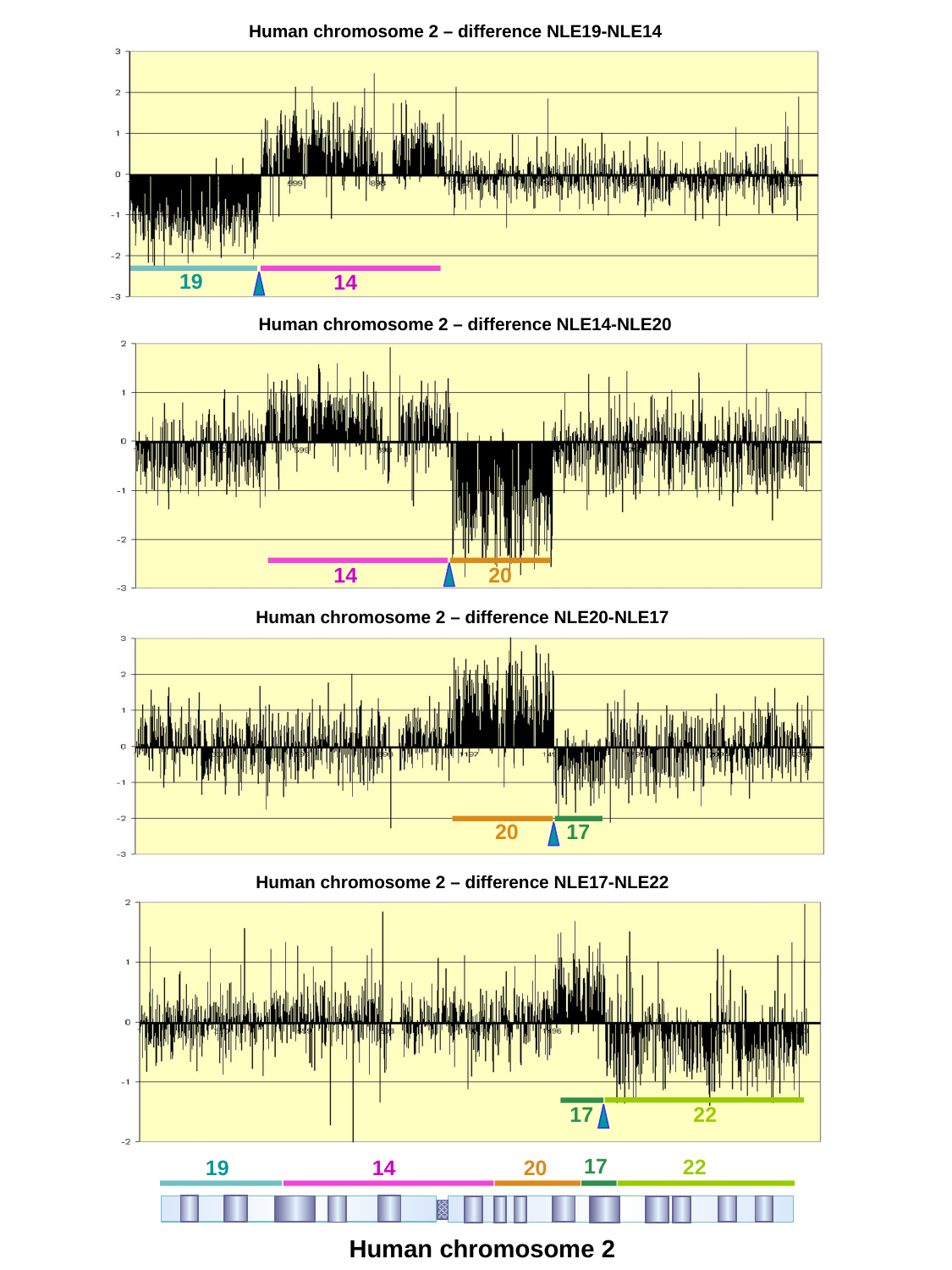

Human chromosome 2 – difference NLE19-NLE14
19
14
Human chromosome 2 – difference NLE14-NLE20
14
20
Human chromosome 2 – difference NLE20-NLE17
17
20
Human chromosome 2 – difference NLE17-NLE22
17
22
17
22
19
14
20
Human chromosome 2

## Slide 3
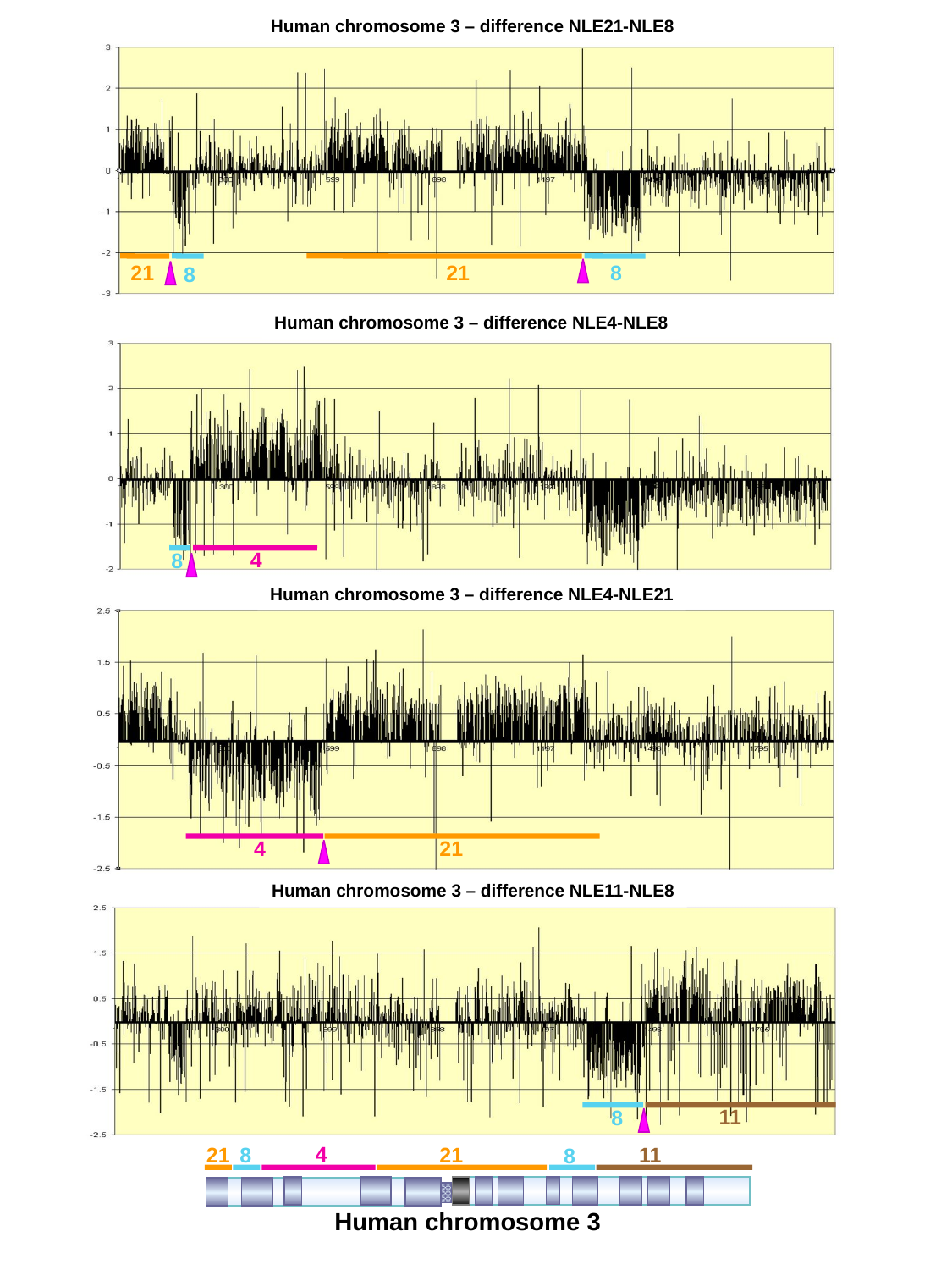

Human chromosome 3 – difference NLE21-NLE8
21
21
8
8
Human chromosome 3 – difference NLE4-NLE8
4
8
Human chromosome 3 – difference NLE4-NLE21
4
21
Human chromosome 3 – difference NLE11-NLE8
11
8
4
11
21
8
21
8
Human chromosome 3

## Slide 4
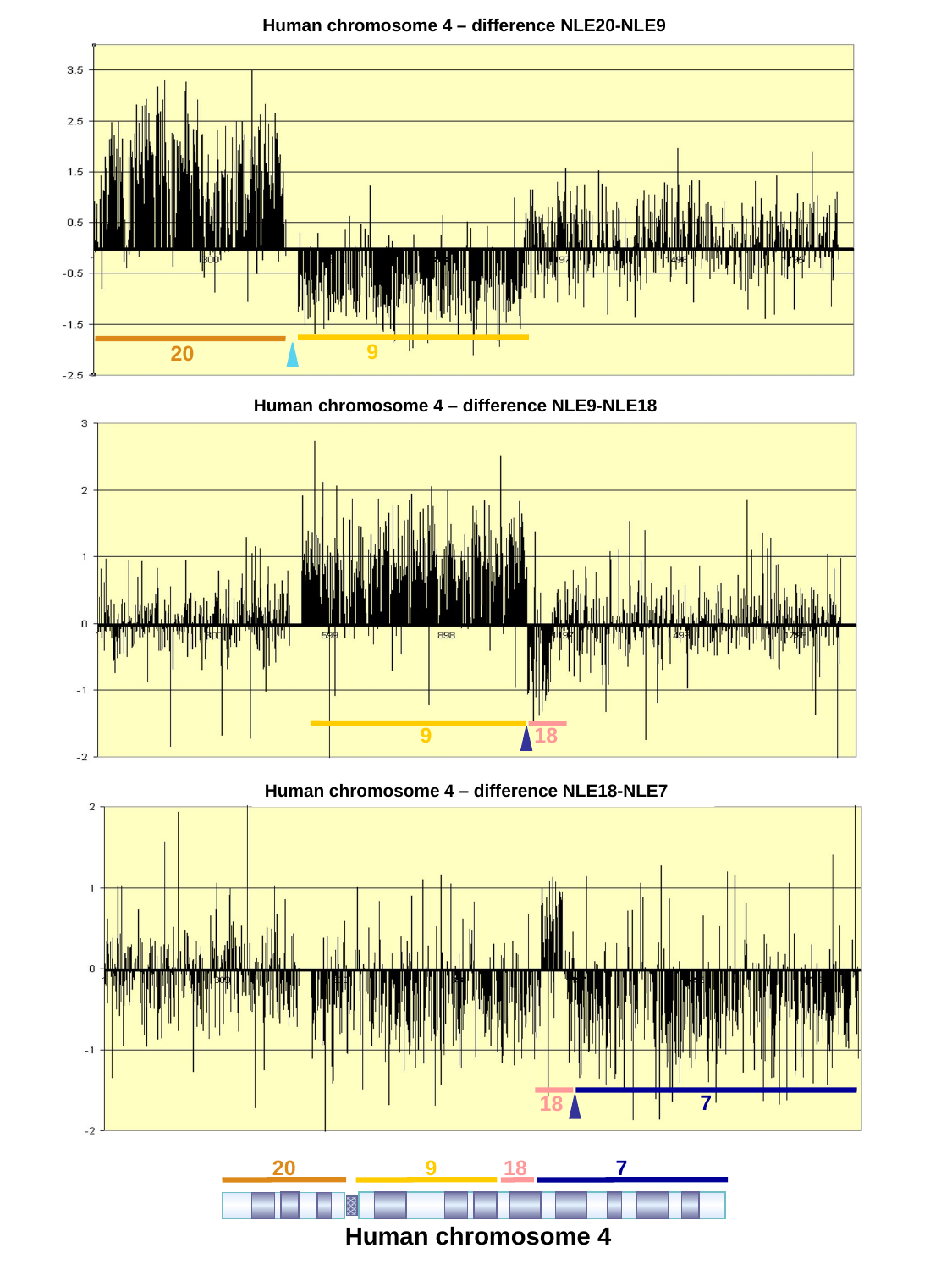

Human chromosome 4 – difference NLE20-NLE9
9
20
Human chromosome 4 – difference NLE9-NLE18
9
18
Human chromosome 4 – difference NLE18-NLE7
7
18
20
18
9
7
Human chromosome 4

## Slide 5
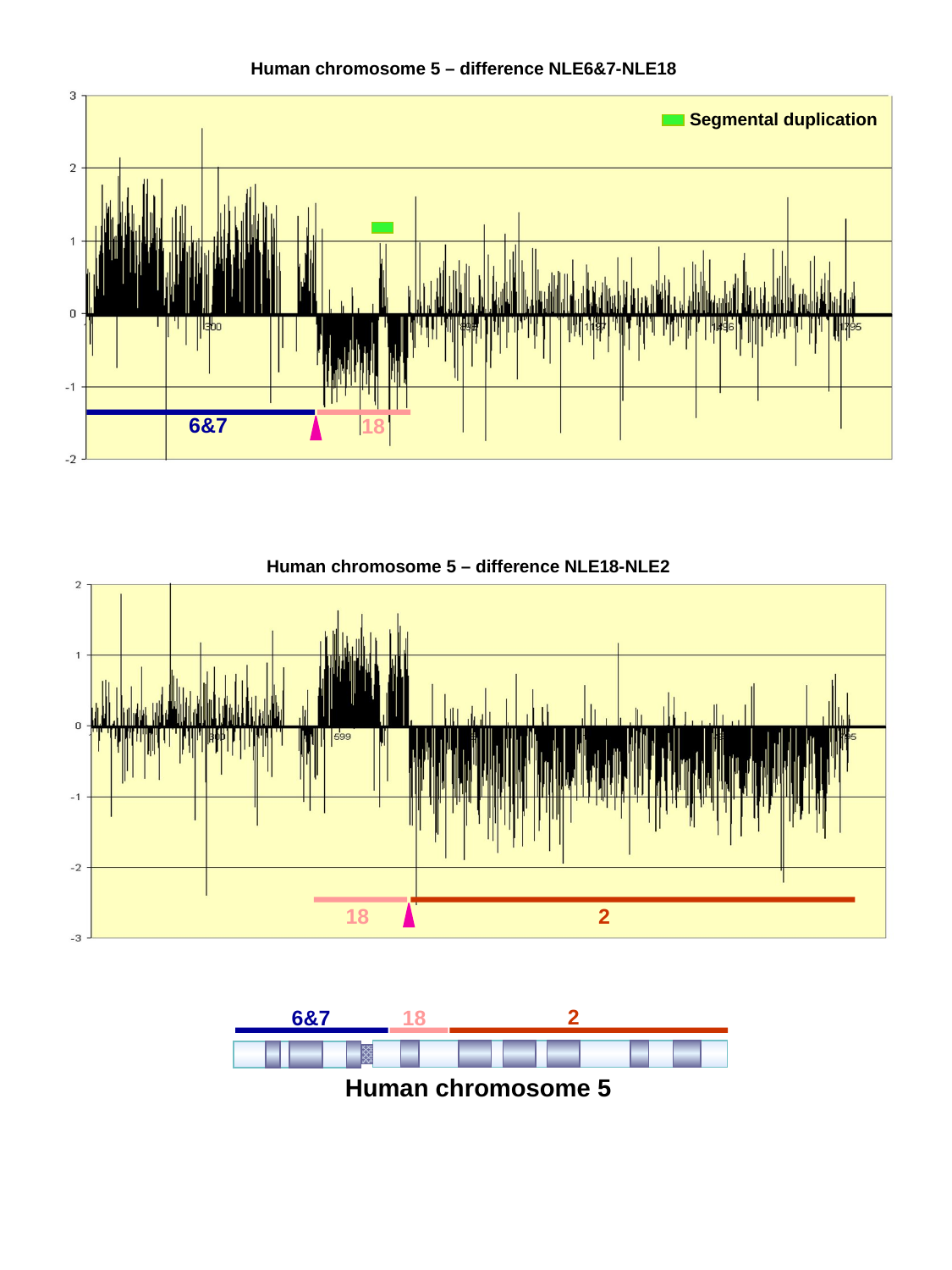

Human chromosome 5 – difference NLE6&7-NLE18
Segmental duplication
6&7
18
Human chromosome 5 – difference NLE18-NLE2
18
2
2
6&7
18
Human chromosome 5

## Slide 6
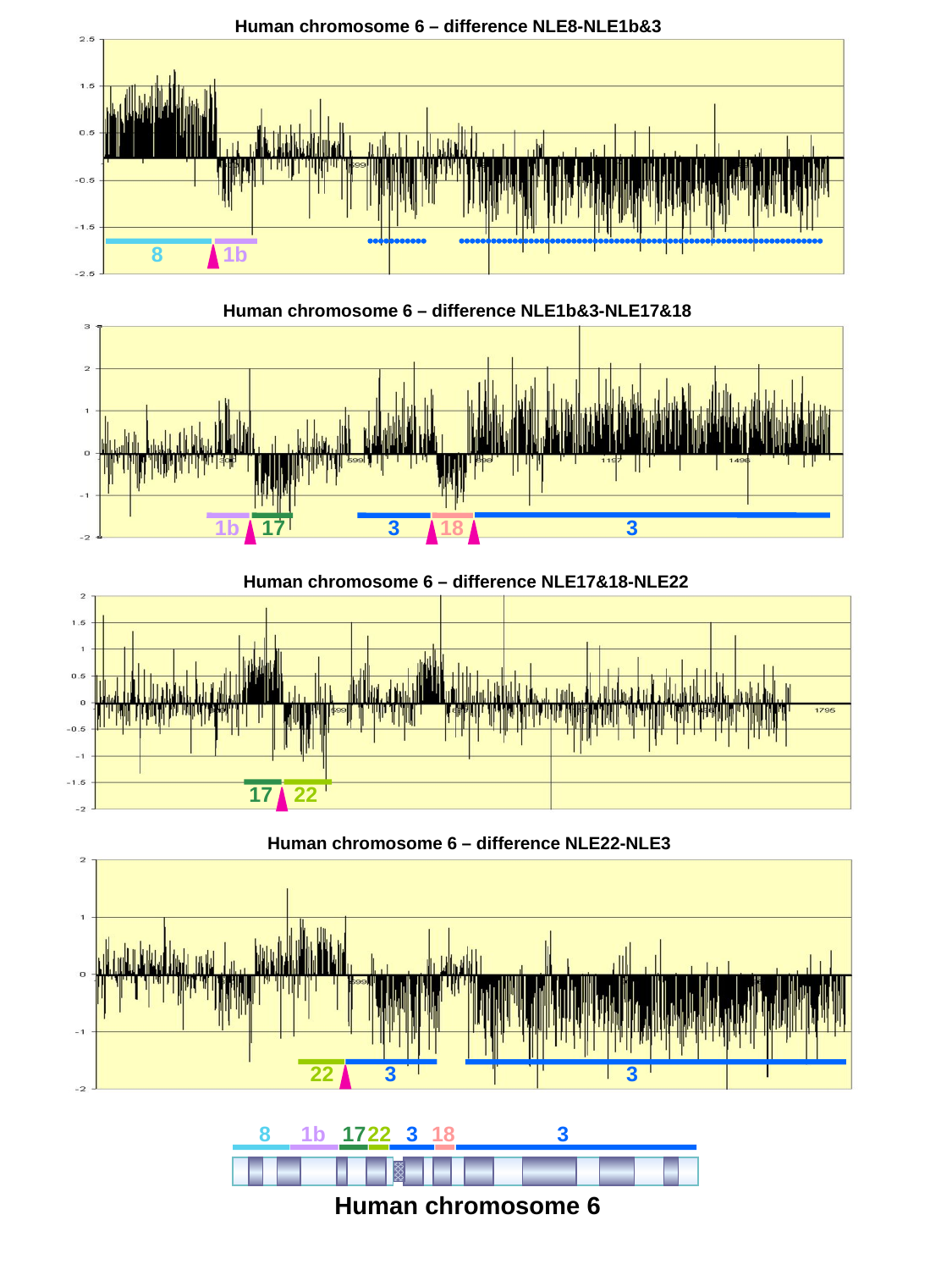

Human chromosome 6 – difference NLE8-NLE1b&3
8
1b
Human chromosome 6 – difference NLE1b&3-NLE17&18
1b
18
17
3
3
Human chromosome 6 – difference NLE17&18-NLE22
17
22
Human chromosome 6 – difference NLE22-NLE3
22
3
3
1b
17
3
8
22
3
18
Human chromosome 6

## Slide 7
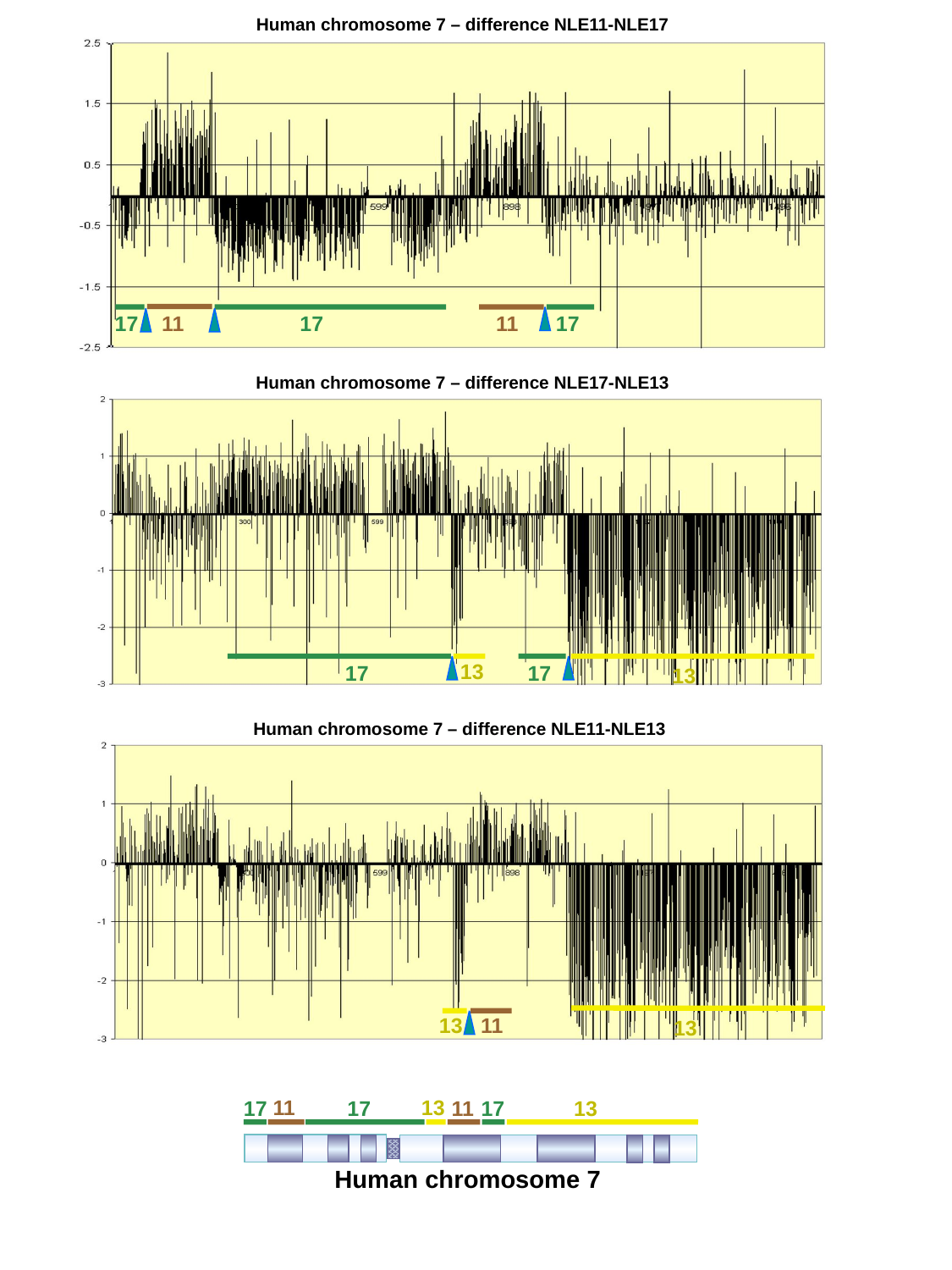

Human chromosome 7 – difference NLE11-NLE17
17
11
17
11
17
Human chromosome 7 – difference NLE17-NLE13
13
17
17
13
Human chromosome 7 – difference NLE11-NLE13
13
11
13
11
13
11
13
17
17
17
Human chromosome 7

## Slide 8
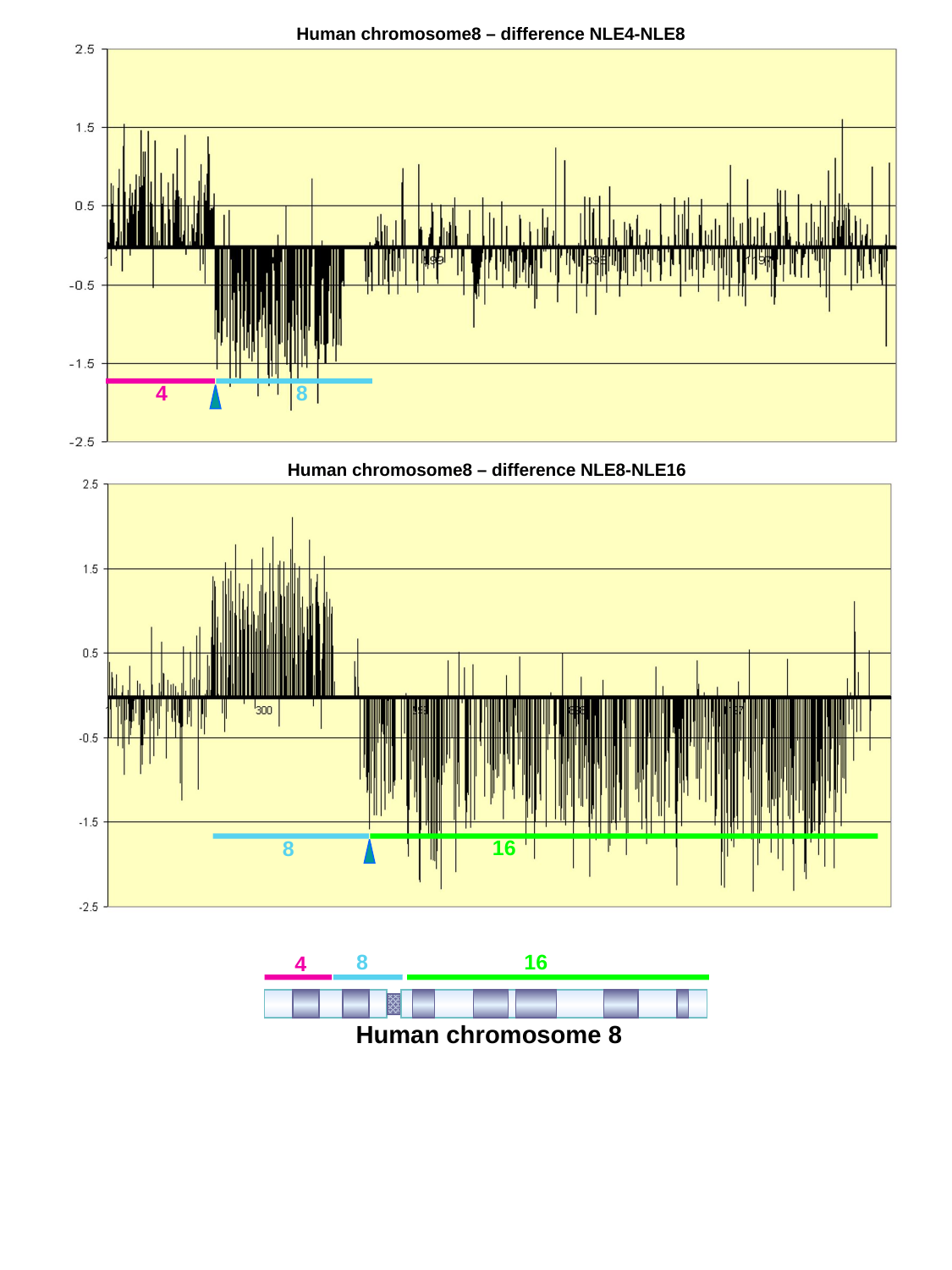

Human chromosome8 – difference NLE4-NLE8
4
8
Human chromosome8 – difference NLE8-NLE16
16
8
8
16
4
Human chromosome 8

## Slide 9
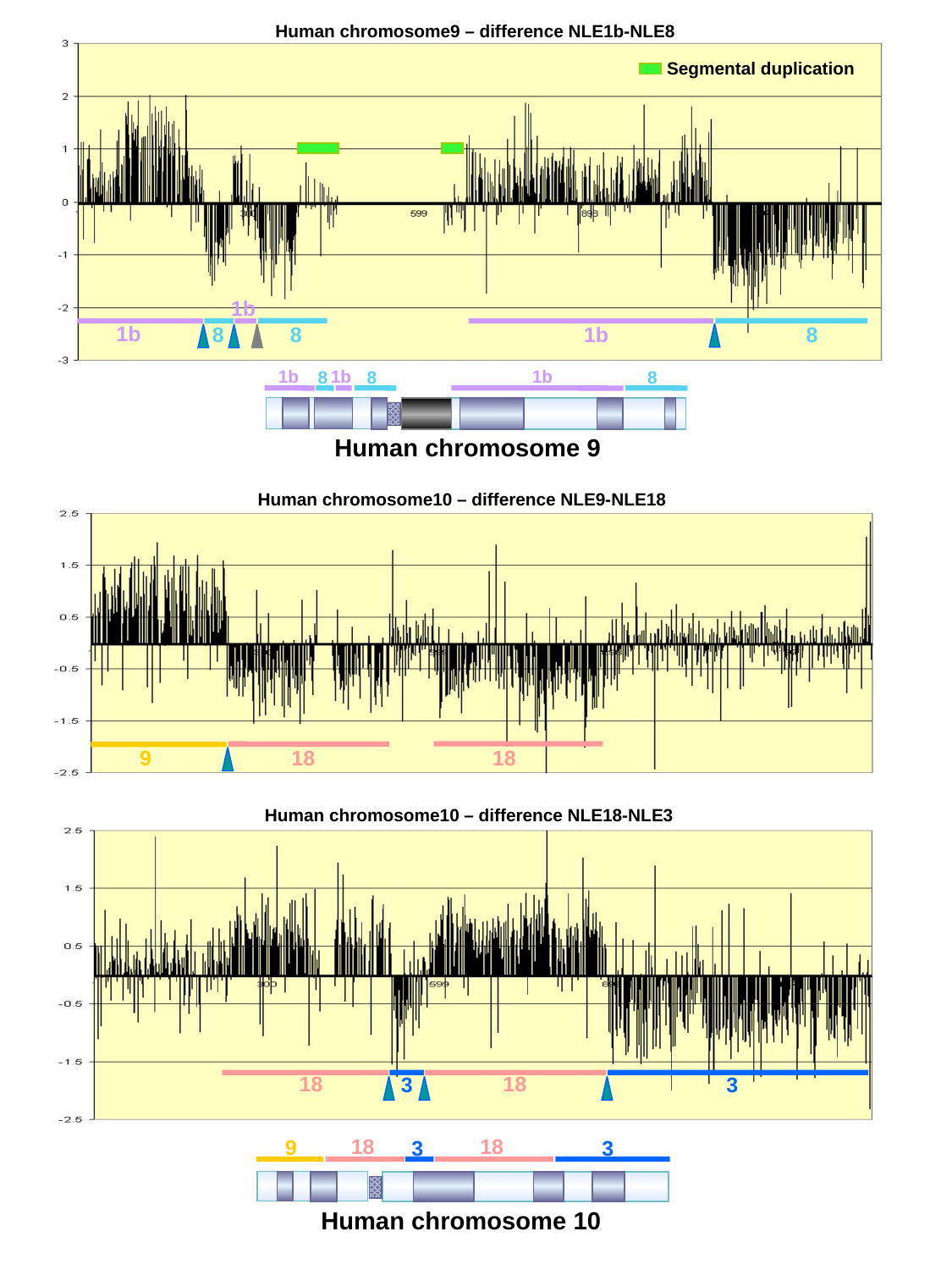

Human chromosome9 – difference NLE1b-NLE8
Segmental duplication
1b
1b
8
8
1b
8
1b
1b
1b
8
8
8
Human chromosome 9
Human chromosome10 – difference NLE9-NLE18
9
18
18
Human chromosome10 – difference NLE18-NLE3
18
18
3
3
18
18
9
3
3
Human chromosome 10

## Slide 10
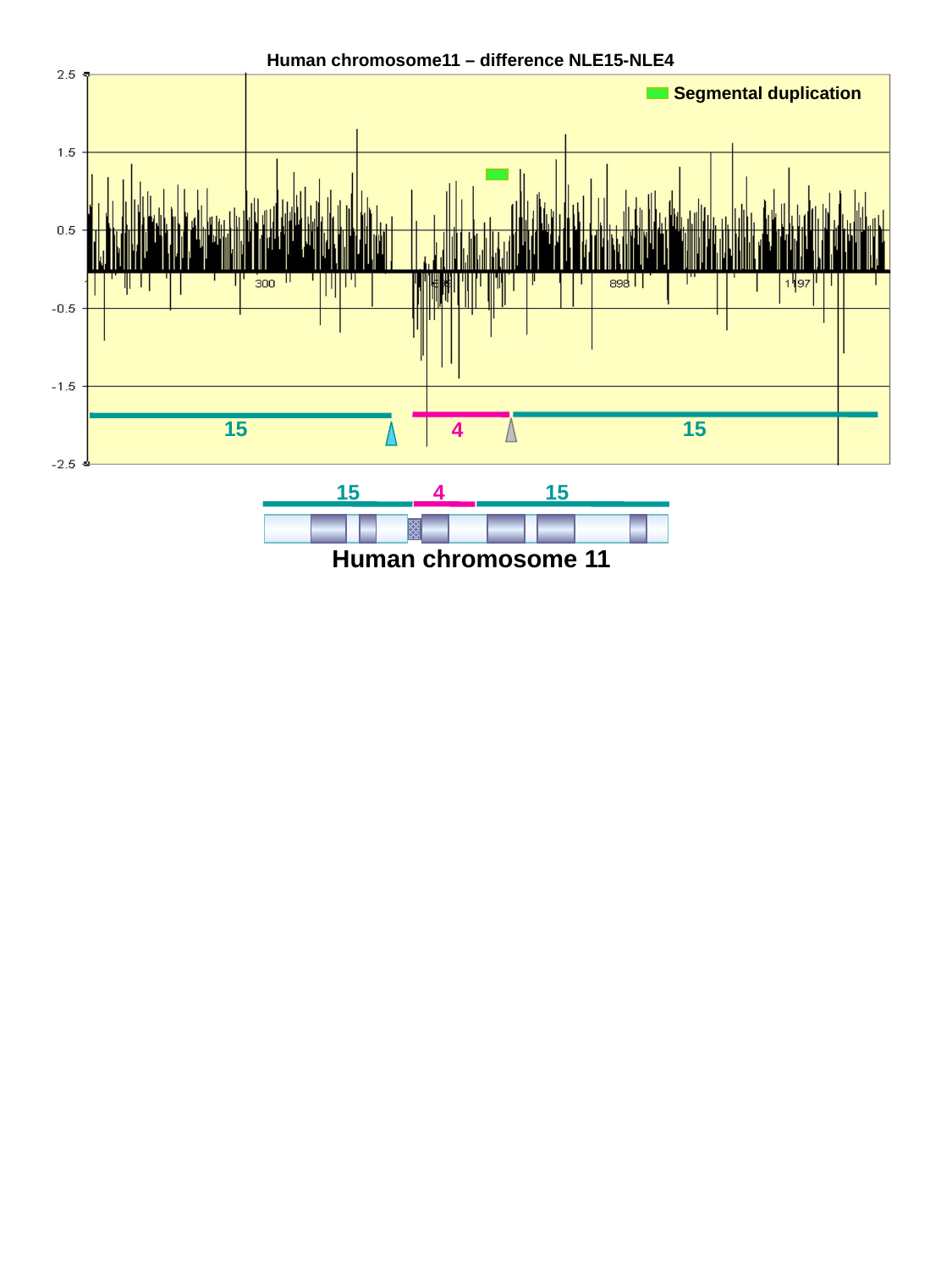

Human chromosome11 – difference NLE15-NLE4
Segmental duplication
15
15
4
15
15
4
Human chromosome 11

## Slide 11
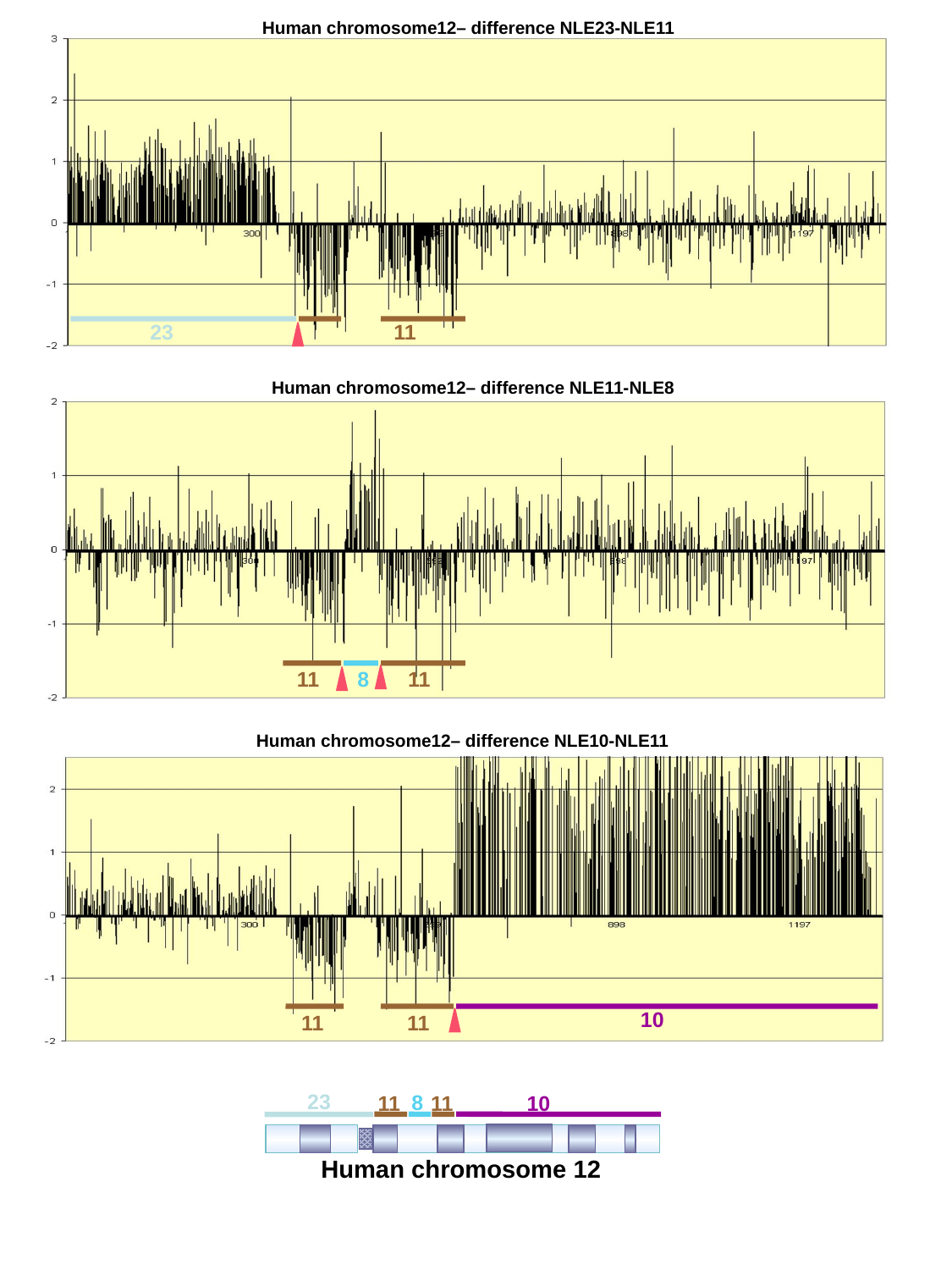

Human chromosome12– difference NLE23-NLE11
23
11
Human chromosome12– difference NLE11-NLE8
11
8
11
Human chromosome12– difference NLE10-NLE11
10
11
11
23
8
11
11
10
Human chromosome 12

## Slide 12
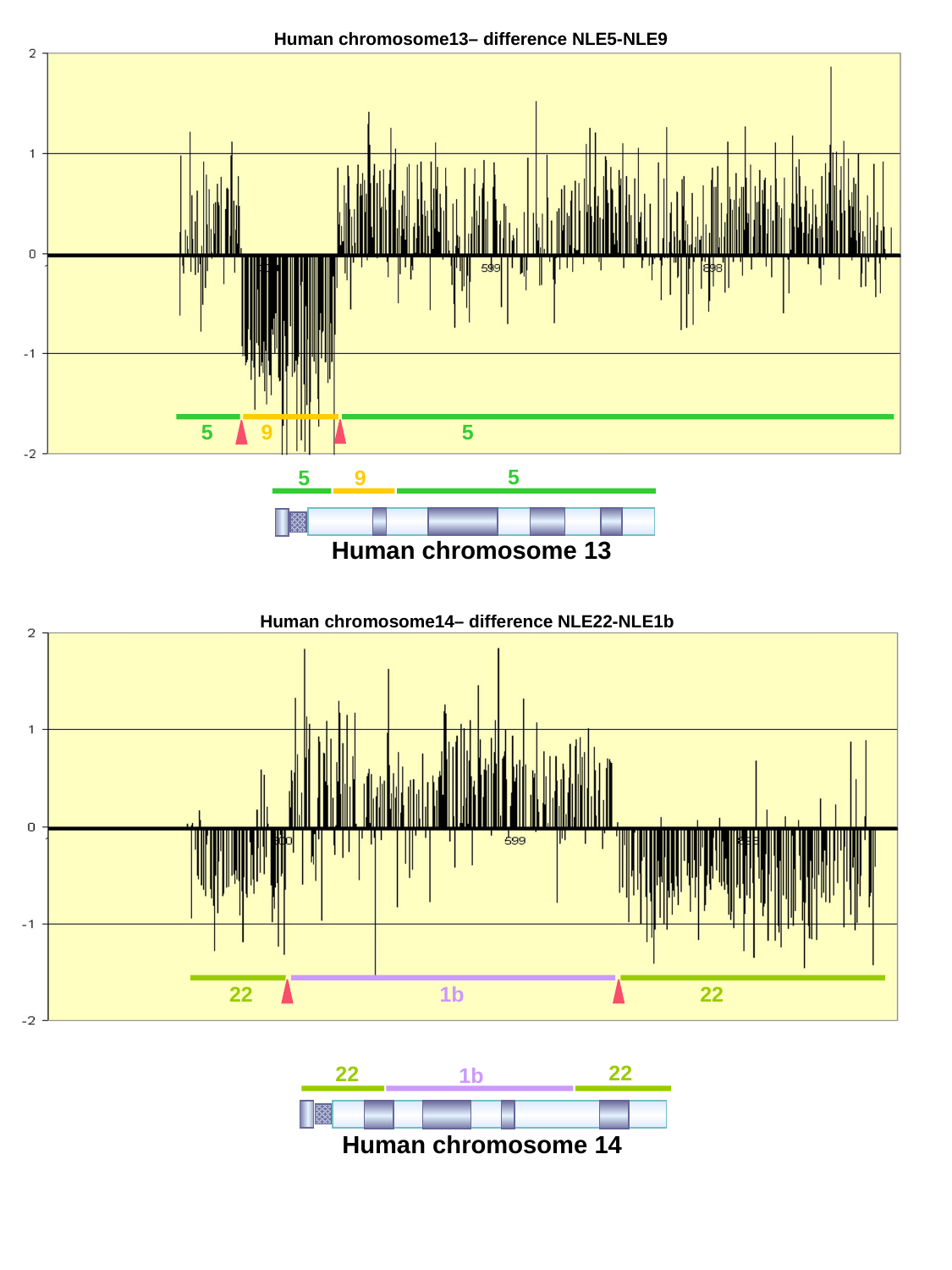

Human chromosome13– difference NLE5-NLE9
5
9
5
5
5
9
Human chromosome 13
Human chromosome14– difference NLE22-NLE1b
22
1b
22
22
22
1b
Human chromosome 14

## Slide 13
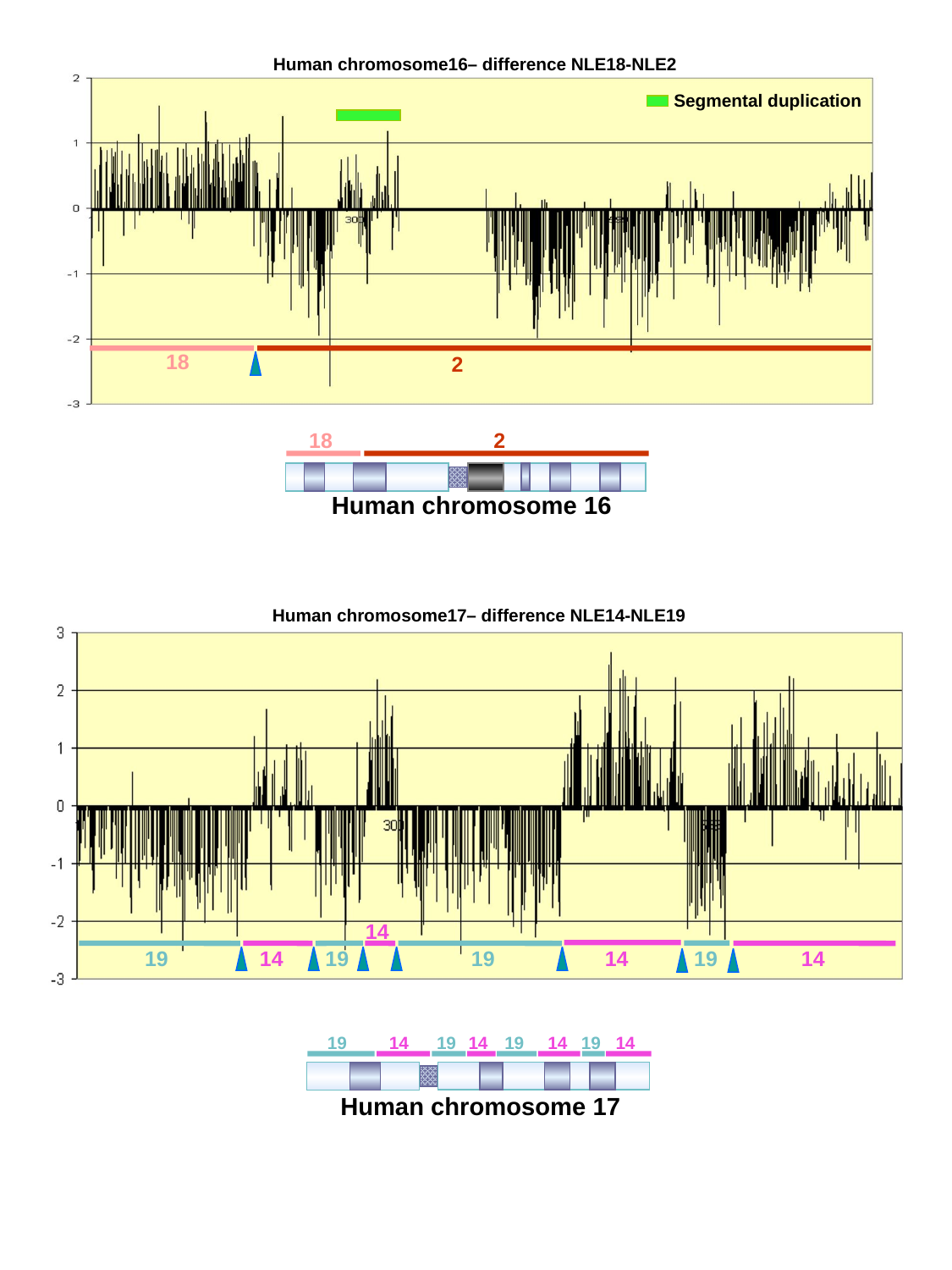

Human chromosome16– difference NLE18-NLE2
Segmental duplication
18
2
18
2
Human chromosome 16
Human chromosome17– difference NLE14-NLE19
14
19
14
19
19
14
19
14
19
14
19
14
19
14
19
14
Human chromosome 17

## Slide 14
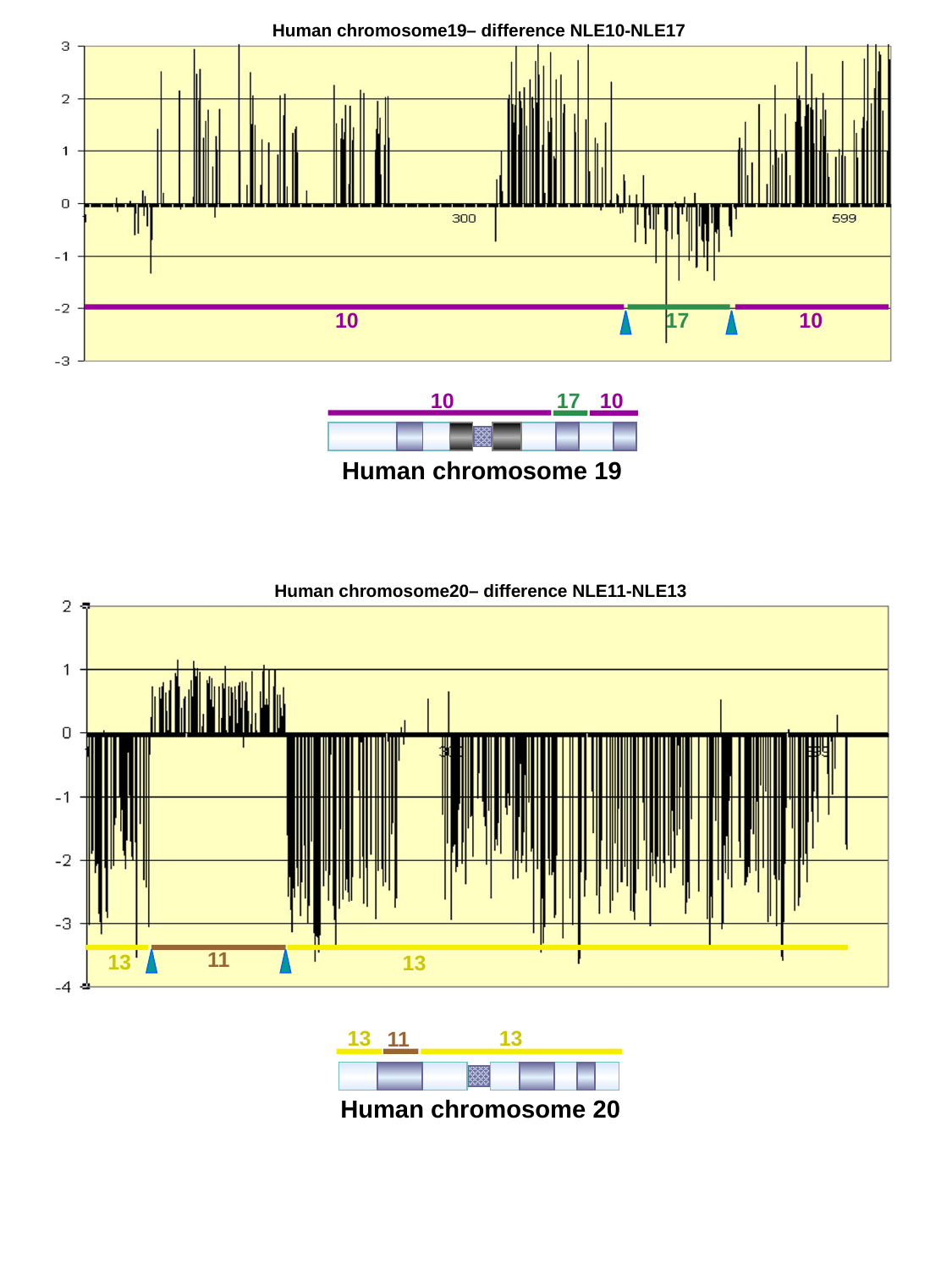

Human chromosome19– difference NLE10-NLE17
10
17
10
10
17
10
Human chromosome 19
Human chromosome20– difference NLE11-NLE13
11
13
13
13
13
11
Human chromosome 20
