# Supplementary figures and images for: A High-Resolution Map of Synteny Disruptions in Gibbon and Human Genomes
Source: PLoS Genet. 2006 Dec 29;2(12):e223. doi: 10.1371/journal.pgen.0020223 (PMC1756914; doi:10.1371/journal.pgen.0020223)

## Slide 1
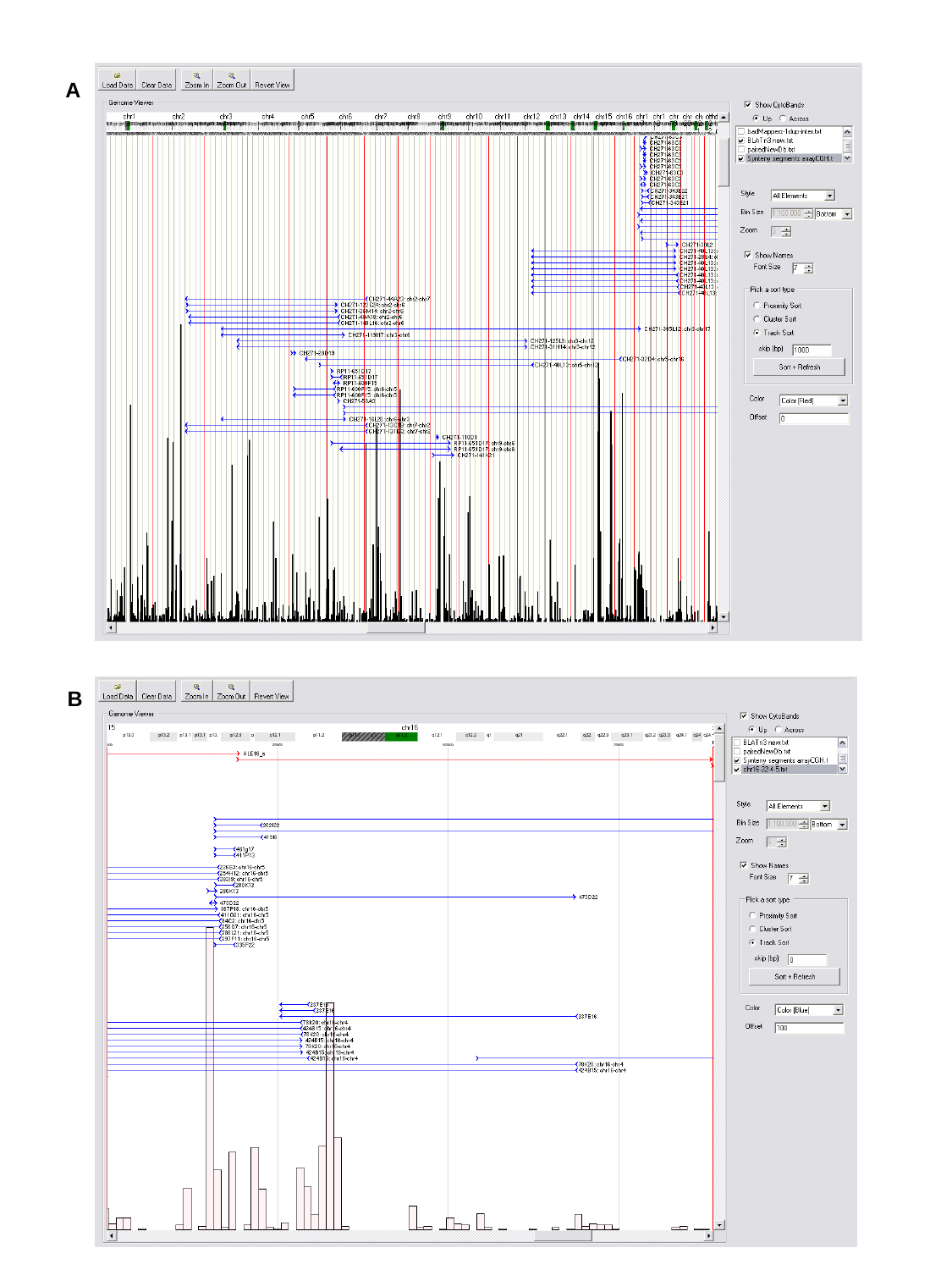

A
B

Supplement: Figure S2 — This tool was developed in order to easily localize gibbon clones spanning a translocation or inversion breakpoint in human. Figure S2A corresponds to a full genome view and Figure S2B corresponds to Chromosome 16. Gibbon clones are represented by the blue arrows, taking into account the orientation of each BES. On the bottom of the window is a density plot of human SDs. (493 KB PPT) [file pgen.0020223.sg002.ppt]

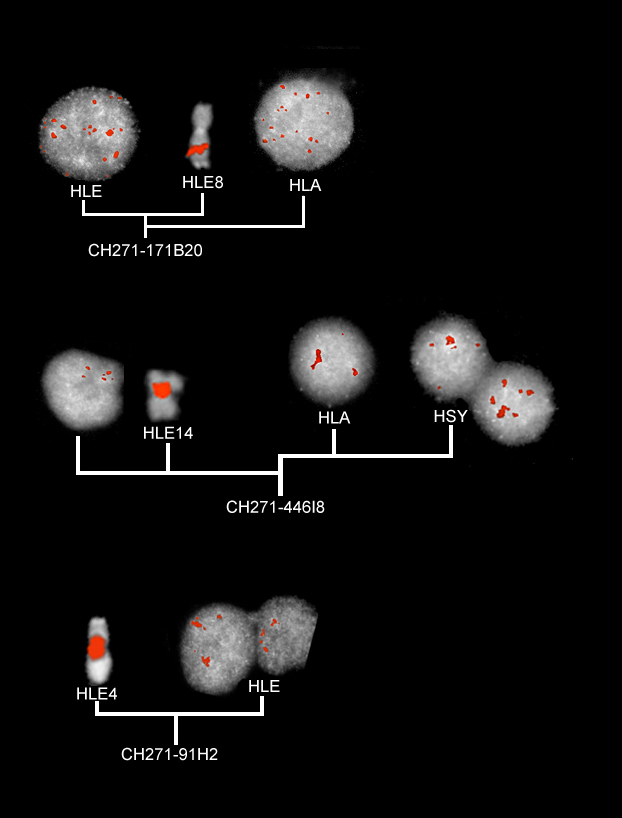

Supplement: Figure S3 — A sample of gibbon BAC clones overlapping human SDs was hybridized on NLE nuclei. The presence of duplications was revealed by the presence of either multiple signals or a single but broadened signal. The figure shows the results obtained with four clones also tested on HLA and S. syndactylus. (122 KB JPG) [file pgen.0020223.sg003.jpg]
