# Supplementary material for: A High-Resolution Map of Synteny Disruptions in Gibbon and Human Genomes
Source: PLoS Genet. 2006 Dec 29;2(12):e223. doi: 10.1371/journal.pgen.0020223 (PMC1756914; doi:10.1371/journal.pgen.0020223)
Supplement: Protocol S1 [file pgen.0020223.sd001.doc]

**Protocol S1**

**NOISE REDUCTION METHOD**

When the hybridization results were analyzed a low signal-to-noise ratio was observed preventing an accurate determination of BOSR coordinates. This effect can be the results of two different trends. The background might be random due to poor, irreproducible, hybridization of the gibbon probe and this may reflect the evolutionary distance between gibbons and humans which results in a higher noise level than when comparing only human samples. Alternatively, the noise could reproduce systematic [non-random] variation due to region-specific divergence between the test and reference probes. The hybridization results obtained with the different gibbon chromosome pools were combined for individual human chromosomes to explore the latter possibility. Pairs of hybridization results for gibbon chromosomes sharing the same BOSR as consequence of a putative reciprocal translocation event were combined by subtracting the test-reference values for the spots corresponding to the syntenic human chromosomes. After applying our method on chromosomes NLE14 and NLE19 we obtained a strikingly low-noise result visible in figure 1c. To demonstrate that the difference method isn’t just an optical effect, we attempted to make measurements of the noise reduction in the arbitrarily chosen case of the NLE14/NLE19 breakpoint boundary. We chose the standard deviation of the data sets as a measure of noise, and compared this measure in the original datasets versus the combined (subtracted) dataset. If the sets exhibited purely stochastic noise the combination of both sets by subtraction would result in an increase of standard deviation and therefore of noise. We observed however that in our case the subtraction method resulted in a decrease of noise: the opposite of the stochastic case. The noise reduction in comparison to the stochastic case was of about 2.1 fold (data not shown). In addition we confirmed that the noise was systematic by measuring the correlation between the 2 original data sets: we measured extremely high correlations 0.77, 0.74 and 0.9 in regions 1, 2 and 3 respectively (see figure 1c).

From these results we concluded that the noise produced during the hybridization was systematic and therefore we could use this effect to obtain better defined images of the breakpoints in all the hybridizations (figure S1).

**GIBBON BAC LIBRARY CONSTRUCTION and BAC End Sequences (BES) MAPPING**

The genomic BAC library CHORI-271 was constructed following the cloning approach developed in our laboratory. The DNA was isolate from blood from a wild-born Northern White-cheeked female “Asia” with international studbook #0098. The blood was kindly provided by Alan Mootnick, Director of the Gibbon Conservation Center (GCC), Santa Clarita, California. Blood samples were collected on May 18th 2004. The average insert size of the library corresponds to 170Kb (32Kb standard deviation), as determined by *Not* I digestion and pulsed-field electrophoresis of a subset of clones. Further information about this library can be found at the web-site <http://bacpac.chori.org/library.php?id=228>.

Both ends of 5376 clones (corresponding to 14x384 wells plates) from the CHORI-271 BAC library were selected for end sequencing. This means that our data set allowed sampling 26.8% of the gibbon genome, and therefore we expect about 25% of the evolutionary breakpoints might be present in this sample. Individual reads were trimmed for high quality sequence using a phred cutoff score of 20 and subsequently mapped onto the human genome (ncbi 35 freeze) using the blat alignment tool. Best forward and best reverse hits were selected based on having an alignment length at least as high as seventy percent the alignment length of the best hit for an end.

**IDENTIFICATION OF CLONES SPANNING PUTATIVE BREAKPOINTS**

After the BES were mapped by BLAT, the output was processed with a perl script that classifies read pairs from a clone as matched, or unmatched. Since the reads can map to more than one location on the genome for each end of each clone, the script inspected one by one each combination of top forward to best reverse hits given by one clone. To call a clone as “matched” we checked for the standard end pairing criteria: a read combination had to map within 3 standard deviations from the average insert size of the library and the two reads had to point towards each other [33]. Reads were called “unmatched” if they did not meet the latter criteria. Unmatched reads mapping to different chromosomes were labeled as candidates for inter-chromosomal rearrangements. Unmatched end reads that mapped within the same chromosome and that did not meet the match criteria were labeled as putative intra-chromosomal rearrangements. Additionally, our script kept track of the quality of the evidence by discerning whether a clone was mapped to a unique location or to multiple sites.

**TRANSLOCATION VIEWER**

In order to investigate how the hits were localized on the human genome, we developed a tool that lets one view the genome and the locations of the clones all at one glance and in a highly interactive manner: the “translocation viewer” was designed using C#. The output of the latter perl script can be fed to the translocation viewer using a tracks system. Using this track system other types of information, such as segmental duplications could be added. Sorting the display proximity of the clones according to different criteria was also possible (figure S2).

**SIMULATIONS FOR STATISTICAL ANALYSIS**

The statistical analysis of the overlap between the 100 BOSRs identified in the study and SD was performed with the help of a C# application written in-house. Tracks of genome wide repeat content, repeat content for different subcategories of repeats, for segmental duplications were prepared for input to the simulation software using data from [http://genome.ucsc.edu](http://genome.ucsc.edu/) human genome May 2005 release (hg17). Subsequently the latter track was used in order to perform different overlap measurements with the set of 100 BOSRs. Three types of measures were adopted: “count 1”, which measured the existence of an element in each region of the set and returned a total count for the set, “count all” which measured the cumulated count of elements from the track overlapping with the regions of the set, and “count bp” which measured the cumulated base pair overlap between the regions and the track. In the first place, these different measurements were used to assess the general landscape in which the breakpoint regions were located. Subsequently the regions were reshuffled randomly in their chromosome of origin, their size being kept the same as the original counterpart, and the same measurements were taken, in order to look at the global genome wide picture. Random reshuffling was reiterated 1000 times and the resulting sampling distribution was plotted to compare the original set of regions with the global genomic landscape.
