# Supplementary material for: A High-Resolution Map of Synteny Disruptions in Gibbon and Human Genomes
Source: PLoS Genet. 2006 Dec 29;2(12):e223. doi: 10.1371/journal.pgen.0020223 (PMC1756914; doi:10.1371/journal.pgen.0020223)
Supplement: Table S1 — The gibbon chromosomes were divided into four pools in order to minimize the number of array-painting experiments. A smart-pooling strategy was used, taking advantage of the data available in the literature. Through this approach the repetition of one human chromosome in the same pool was avoided. Additionally, three gibbon chromosomes were hybridized in individual experiments. (45 KB DOC) [file pgen.0020223.st001.doc]

| **POOL** | ***N.leucogenys* chromosomes** | ***Homo sapiens* chromosomes** |
| --- | --- | --- |
| **A** | 18 | **6q**/10/4/5/16 |
|  | 15/12 | 11/1 |
|  | 23 | 12 |
|  | 17 | **6p**/2/7/19 |
|  | 21 | 3 |
| **B** | 11/9 | 20/7/12/3 |
|  | 16 | 8 |
|  | 22(b) | 14/6/2 |
|  | 6/7 | 5/15/22/4/5 |
| **C** | 19 | 17/2 |
|  | 8 | 12/3/8/6/9 |
|  | 9 | 13/**1q**/4/10 |
|  | 24 | **1p** |
| **D** | 1(b) | 9/**6p**/14 |
|  | 2 | 5/16 |
|  | 3 | 10/**6q** |
|  | 4 | 18/11/3/8 |
|  | 5/X | 1/13 |
|  | 14 | 2/17 |
| **Single** |  | |
| **E** | 13 | 7/20 |
| **F** | 20 | 2/4 |
| **G** | 10 | 12/19 |

**Table S1**
