# Supplementary material for: A High-Resolution Map of Synteny Disruptions in Gibbon and Human Genomes
Source: PLoS Genet. 2006 Dec 29;2(12):e223. doi: 10.1371/journal.pgen.0020223 (PMC1756914; doi:10.1371/journal.pgen.0020223)
Supplement: Table S2 — The table reports the outcome of the mapping of gibbon clones spanning BOSRs on the latest genome assembly of Rhesus macaque (reMach2) and chimpanzee (panTro1). Depending on the result, clones were classified into three evolutionary groups: 1) gibbon specific, 2) great ape specific, and 3) human specific. Mapping results not consistent with human are in italics. (134 KB DOC) [file pgen.0020223.st002.doc]

|  | HUMAN | | CHIMPANZEE | | RHESUS MACAQUE | |  |
| --- | --- | --- | --- | --- | --- | --- | --- |
| **Clone** | **Human chromosome(s)** | **Rearrangement**  **type** | **Chimpanzee**  **chromosome(s)** | **Rearrangement**  **type** | **Rhesus**  **Chromosome(s)** | **Rearrangement**  **type** | **EVOLUTIONARY GROUP** |
| CH271-114O8 | 5-16 | Interchromosomal | 4random-18 | Interchromosomal | 6-20 | Interchromosomal | **Gibbon Specific** |
| CH271-122E24 | 2-6 | Interchromosomal | 5-13 | Interchromosomal | 4-12 | Interchromosomal | **Gibbon Specific** |
| CH271-125L9 | 3-12 | Interchromosomal | 2-10 | Interchromosomal | 2-11 | Interchromosomal | **Gibbon Specific** |
| CH271-141K21 | 9 | Intrachromosomal | 11 | Intrachromosomal | 15 | Intrachromosomal | **Gibbon Specific** |
| CH271-171B20 | 9 | Intrachromosomal | 11 | Intrachromosomal | 15 | Intrachromosomal | **Gibbon Specific** |
| CH271-185K6 | 14 | Intrachromosomal | 15/15 random | Intrachromosomal | 7 | Intrachromosomal | **Gibbon Specific** |
| CH271-226E3 | 5-16 | Interchromosomal | 4-18 | Interchromosomal | 6-20 | Interchromosomal | **Gibbon Specific** |
| CH271-228C01  CH271-275I11 | 7-20 | Interchromosomal | 6-21 | Interchromosomal | 3-10 | Interchromosomal | **Gibbon Specific** |
| CH271-234B14 | 3-8 | Interchromosomal | 2-7 | Interchromosomal | 2-8 | Interchromosomal | **Gibbon Specific** |
| CH271-237A9 | 12-19 | Interchromosomal | 10-20 | Interchromosomal | 11-19 | Interchromosomal | **Gibbon Specific** |
| CH271-247C2 | 2-17 | Interchromosomal | 11-19 | Interchromosomal | 13-16 | Interchromosomal | **Gibbon Specific** |
| CH271-262O07 | 5-8 | Interchromosomal | 4-7 | Interchromosomal | 6-8 | Interchromosomal | **Gibbon Specific** |
| CH271-183B5 | 5-8 | Interchromosomal | 4-7 | Interchromosomal | NA |  | **Gibbon Specific** |
| CH271-263C09 | 4-22 | Interchromosomal | 3-23random | Interchromosomal | 5-10 | Interchromosomal | **Gibbon Specific** |
| CH271-267G23 | 2-17 | Interchromosomal | 12-19random | Interchromosomal | 13-16 | Interchromosomal | **Gibbon Specific** |
| CH271-269N07 | 2-6 | Interchromosomal | 5-13 | Interchromosomal | 4-12 | Interchromosomal | **Gibbon Specific** |
| CH271-274B24 | 14-6 | Interchromosomal | 5-15 | Interchromosomal | 4-7 | Interchromosomal | **Gibbon Specific** |
| CH271-275P15 | 7 | Intrachromosomal | 6 | Intrachromosomal | 3 | Intrachromosomal | **Gibbon Specific** |
| CH271-350B17 | 4-5 | Interchromosomal | 3-4 | Interchromosomal | 5-6 | Interchromosomal | **Gibbon Specific** |
| CH271-380N5 | 3-12 | Interchromosomal | 2-10 | Interchromosomal | 2-11 | Interchromosomal | **Gibbon Specific** |
| CH271-393O10 | 17 | Intrachromosomal | 19 | Intrachromosomal | 16 | Intrachromosomal | **Gibbon Specific** |
| CH271-401L9 | 4-10 | Interchromosomal | 3-8 | Interchromosomal | 5-9 | Interchromosomal | **Gibbon Specific** |
| CH271-405A9 | 2-17 | Interchromosomal | 12-19random | Interchromosomal | 13-16 | Interchromosomal | **Gibbon Specific** |
| CH271-40A18 | 2-6 | Interchromosomal | 5-13 | Interchromosomal | 4-12 | Interchromosomal | **Gibbon Specific** |
| CH271-133C19 | 17 | Intrachromosomal | 19 | Intrachromosomal | 16 | No Rearrangement | **Great Apes Specific** |
| CH271-261A22 | 7 | Intrachromosomal | 6 | Intrachromosomal | 3 | No Rearrangement | **Great Apes Specific** |
| CH271-263D23 | 1 | Intrachromosomal | 1 | Intrachromosomal | 1 | No Rearrangement | **Great Apes Specific** |
| CH271-202N111 | 10 | Intrachromosomal | 8 | No Rearrangement | 9 | No Rearrangement | **Human Specific** |
| CH271-229B20 | 1 | Intrachromosomal | 1 | No Rearrangement | 1 | No Rearrangement | **Human Specific** |
| CH271-163A2 | 9 | Intrachromosomal | NA |  | 15 | Intrachromosomal | **Gibbon Specific** |
| CH271-228N13 | 10 | Intrachromosomal | 8 | Intrachromosomal | 9 | Intrachromosomal | **Gibbon Specific** |
|  | HUMAN | | CHIMPANZEE | | RHESUS MACAQUE | |  |
| **Clone** | **Human chromosome(s)** | **Rearrangement**  **type** | **Chimpanzee**  **chromosome(s)** | **Rearrangement**  **type** | **Rhesus**  **Chromosome(s)** | **Rearrangement**  **type** | **EVOLUTIONARY GROUP** |
| CH271-262E11 | 2-17 | Interchromosomal | *12-17* | Interchromosomal | 13-16 | Interchromosomal | **Gibbon Specific** |
| CH271-262G05 | 2-17 | Interchromosomal | 12-19 | Interchromosomal | *13-14* | Interchromosomal | **Gibbon Specific** |
| CH271-269B20 | 5-22 | Interchromosomal | *1-4* | Interchromosomal | NA |  | **Gibbon Specific** |
| CH271-269J08 | 16-22 | Interchromosomal | 18random-23 | Interchromosomal | NA |  | **Gibbon Specific** |
| CH271-270H22 | 4-2 | Interchromosomal | 3-13 | Interchromosomal | 5-16 | Interchromosomal | **Gibbon Specific** |
| CH271-274L01 | 17 | Intrachromosomal | 19 | Intrachromosomal | NA |  | **Gibbon Specific** |
| CH271-286K22 | 7 | Intrachromosomal | 6 | Intrachromosomal | NA |  | **Gibbon Specific** |
| CH271-44A23 | 2-7 | Interchromosomal | *1-13* | Interchromosomal | 3-12 | Interchromosomal | **Gibbon Specific** |
| CH271-267G21 | 17 | Intrachromosomal | NA |  | 16 | No Rearrangement | **Human Specific** |
| CH271-186A1 | 6 | Intrachromosomal | 5 | Intrachromosomal | 4 | Intrachromosomal | **Gibbon Specific** |
| CH271-219C17 | 17 | Intrachromosomal | 19-19random | Intrachromosomal | NA |  | **Gibbon Specific** |
| CH271-246M2 | 2-17 | Interchromosomal | NA |  | 13-16 | Interchromosomal | **Gibbon Specific** |
| CH271-261K06 | 3 | Intrachromosomal | *1-2* | *Interchromosomal* | 11 | No Rearrangement | ***Great Apes Specific*** |
| CH271-261L01 | 1 | Intrachromosomal | 1 | Intrachromosomal | 1 | Intrachromosomal | **Gibbon Specific** |
| CH271-262E15 | 4 | Intrachromosomal | *3-8* | *Interchromosomal* | 5 | Intrachromosomal | **Gibbon Specific** |
| CH271-263M06 | 7 | Intrachromosomal | 6 | Intrachromosomal | NA |  | **Gibbon Specific** |
| CH271-26L7 | 17 | Intrachromosomal | *9-19* | *Interchromosomal* | 16 | Intrachromosomal | **Gibbon Specific** |
| CH271-274K2 | 2 | Intrachromosomal | 13 | Intrachromosomal | *8-13* | *Interchromosomal* | **-** |
| CH271-398E1 | 17 | Intrachromosomal | *1-19* | *Interchromosomal* | 16 | Intrachromosomal | **Gibbon Specific** |

**Table S2**
